# Role of ventral subiculum neuronal ensembles in incubation of oxycodone craving after electric barrier–induced voluntary abstinence

**DOI:** 10.1126/sciadv.add8687

**Published:** 2023-01-11

**Authors:** Ida Fredriksson, Pei-Jung Tsai, Aniruddha Shekara, Ying Duan, Sarah V. Applebey, Angelica Minier-Toribio, Ashley Batista, Jonathan J. Chow, Lindsay Altidor, Estelle Barbier, Carlo Cifani, Xuan Li, David J. Reiner, F. Javier Rubio, Bruce T. Hope, Yihong Yang, Jennifer M. Bossert, Yavin Shaham

**Affiliations:** ^1^ Behavioral Neuroscience Branch, IRP/NIDA/NIH, Baltimore, MD, USA.; ^2^ Center for Social and Affective Neuroscience, Linköping University, Linköping, Sweden.; ^3^ Neuroimaging Research Branch, IRP/NIDA/NIH, Baltimore, MD, USA.; ^4^ School of Pharmacy, University of Camerino, Camerino, Italy.; ^5^ Department of Psychology, University of Maryland College Park, College Park, MD, USA.

## Abstract

High relapse rate is a key feature of opioid addiction. In humans, abstinence is often voluntary due to negative consequences of opioid seeking. To mimic this human condition, we recently introduced a rat model of incubation of oxycodone craving after electric barrier–induced voluntary abstinence. Incubation of drug craving refers to time-dependent increases in drug seeking after cessation of drug self-administration. Here, we used the activity marker Fos, muscimol-baclofen (GABAa + GABAb receptor agonists) global inactivation, Daun02-selective inactivation of putative relapse-associated neuronal ensembles, and fluorescence-activated cell sorting of Fos-positive cells and quantitative polymerase chain reaction to demonstrate a key role of vSub neuronal ensembles in incubation of oxycodone craving after voluntary abstinence, but not homecage forced abstinence. We also used a longitudinal functional magnetic resonance imaging method and showed that functional connectivity changes in vSub-related circuits predict opioid relapse after abstinence induced by adverse consequences of opioid seeking.

## Teaser

A key role of the ventral subiculum in incubation of opioid craving after abstinence induced by adverse consequences.

## INTRODUCTION

High relapse rates are a major contributor to the opioid crisis (*1*). In humans, drug relapse and craving can occur after reexposure to cues and contexts previously associated with drug use (*2*–*4*). In laboratory rats, opioid seeking progressively increases or incubates after homecage forced abstinence from heroin (*5*, *6*) and oxycodone (*7*, *8*) self-administration. Over the past two decades, mechanistic studies have primarily focused on incubation of psychostimulant craving (*9*–*12*). In contrast, the brain mechanisms of incubation of opioid craving are largely unknown (*13*).

In the classical incubation of craving rat model, drug seeking is assessed after homecage forced abstinence (*14*, *15*). This contrasts with the human condition where abstinence is often voluntary and typically involves conflict situations where a person who is using a drug chooses between experiencing the drug’s rewarding effect and the potential adverse consequences of drug use (*16*, *17*). On the basis of these considerations, we recently developed a rat model of incubation of craving after voluntary abstinence, achieved by introducing an “electric barrier” near the drug-paired lever that the rats must cross to gain access to the self-administered drug. Using this model, we reported that oxycodone seeking is higher in the relapse tests after 15 and 30 abstinence days than after 1 day, demonstrating incubation of oxycodone craving after electric barrier–induced abstinence (*7*, *18*). Unexpectedly, in both sexes, incubation of oxycodone craving was stronger after voluntary abstinence than after homecage forced abstinence (*7*).

The goal of the present study was to determine the role of ventral subiculum (vSub) in incubation of oxycodone craving after electric barrier–induced voluntary abstinence. We focused on the vSub because our previous studies indicate a role of vSub in relapse to alcohol seeking after punishment-induced abstinence (*19*) and context-induced reinstatement of heroin seeking (*20*, *21*). There is also evidence for a role of vSub in rats’ behavior in approach-avoidance conflict tasks (*22*, *23*).

We first used immunohistochemistry to measure the neuronal activity marker Fos (*24*) to assess whether incubation of oxycodone craving after electric barrier–induced abstinence is associated with increased activity in vSub. Next, we used the muscimol-baclofen [γ-aminobutyric acid type A (GABAa) and type B (GABAb) receptor agonists] reversible inactivation procedure (*25*) to determine the general role of vSub in incubation of oxycodone craving after either electric barrier–induced abstinence or forced abstinence. Then, we used the Daun02 inactivation method (*26*) to determine the specific role of vSub neuronal ensembles in incubation of oxycodone craving after electric barrier–induced abstinence. This method was developed to study the causal role of Fos-expressing neuronal ensembles in learned behaviors (*26*, *27*). In *Fos-lacZ* transgenic rats (*28*), both Fos and beta-galactosidase (β-gal) are induced in neurons that are strongly activated during learned behaviors. The prodrug Daun02 is injected into discrete brain areas 90 min later. β-Gal converts Daun02 into daunorubicin only in the activated neurons, which inactivates and induces apoptosis in these neurons (*29*, *30*). We and others have previously used the Daun02 inactivation method to assess causal roles of neuronal ensembles in relapse-related behaviors and conditioned drug effects (*30*–*36*). Next, we used fluorescence-activated cell sorting (FACS) (*37*, *38*) and quantitative polymerase chain reaction (qPCR) to assess gene expression in Fos-positive vSub neurons that were activated during incubated oxycodone seeking after electric barrier–induced abstinence and in Fos-negative neurons that represent the nonactivated majority of vSub neurons. We have previously used FACS in combination with qPCR to characterize the molecular phenotype of activated neuronal ensembles in our relapse-related studies (*39*–*41*).

Last, we recently used a rat functional magnetic resonance imaging (fMRI) method (*42*, *43*) and showed that longitudinal resting-state functional connectivity changes of orbitofrontal cortex (OFC) with dorsal striatum and related circuits predict incubation of oxycodone craving after electric barrier–induced abstinence (*18*). Here, we used the brain images from this study to determine whether vSub-related longitudinal functional connectivity changes would also predict this incubation.

## RESULTS

In the experiments described below, we used a rat model of incubation of oxycodone craving after electric barrier–induced voluntary abstinence (*7*). The experiments included some or all of the following phases: oxycodone self-administration training (14 days), early tests for oxycodone seeking (abstinence day 1), electric barrier–induced abstinence or homecage forced abstinence (13 or 16 days), and late tests for oxycodone seeking (abstinence day 15 or 18). We used both male and female rats in experiments 1 to 5 but did not use sex as a factor in the “Statistical analyses” section described below and in table S1, because in our previous study, which was statistically powered to detect sex differences, we did not observe sex differences in oxycodone self-administration, electric barrier–induced abstinence, or incubation of oxycodone craving (*7*).

### Oxycodone self-administration and electric barrier–induced voluntary abstinence

**Fig. 1.**
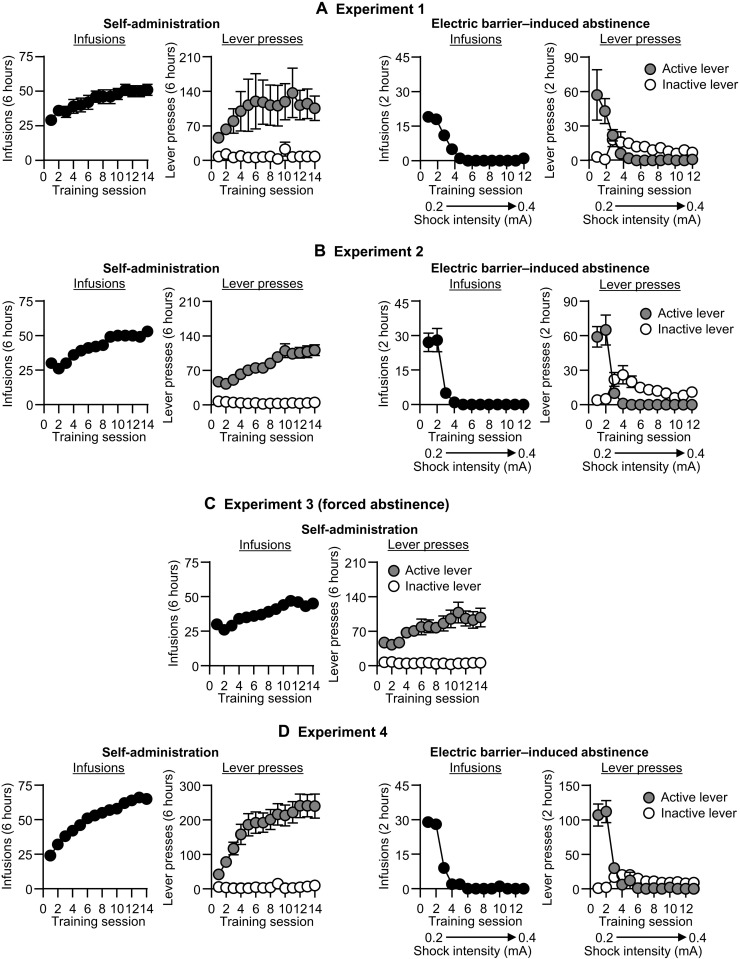
Oxycodone self-administration and electric barrier–induced abstinence (experiments 1 to 4). (**A-D**) Left: Self-administration training. Means ± SEM number of infusions and active and inactive lever presses during the training phase in experiments 1 to 4 (total *n* = 13, 48, 34, and 59, respectively). (A-D) Right: Electric barrier–induced abstinence. Means ± SEM number of infusions and active and inactive lever presses during the electric barrier phase in experiments 1, 2, and 4.

The male and female rats demonstrated reliable oxycodone self-administration (Fig. 1, A to D, and fig. S4B), as indicated by a significant increase in the number of infusions and active lever presses over the training days. The complete analyses for number of infusions and active and inactive lever presses during training are described in table S1.

All rats voluntarily abstained from drug self-administration when we introduced an electric barrier of increasing shock intensity near the active lever, as indicated by a significant decrease in the number of infusions and active lever presses during the abstinence phase (Fig. 1, A, B, and D, and fig. S4B). The mean number of infusions for the last 3 days of electric barrier–induced abstinence was less than one per session. The statistical analyses of the electric barrier–induced abstinence phase are described in table S1. Below, we describe the results of the relapse (incubation) tests of experiments 1 to 5 and then describe the results of the images analyses of experiment 6.

### Incubated oxycodone seeking after electric barrier–induced abstinence is associated with increased vSub activity

The goal of experiment 1 was to determine whether incubation of oxycodone seeking after electric barrier–induced abstinence is associated with increased neuronal activity (assessed by Fos expression) in vSub. For this purpose, we tested male and female rats for oxycodone seeking under extinction conditions 1 day after oxycodone self-administration training and then tested them again after 15 days of electric barrier–induced abstinence. We perfused rats either immediately after the day 15 test (test condition) or the following day (no-test condition).

#### Relapse (incubation) test

**Fig. 2.**
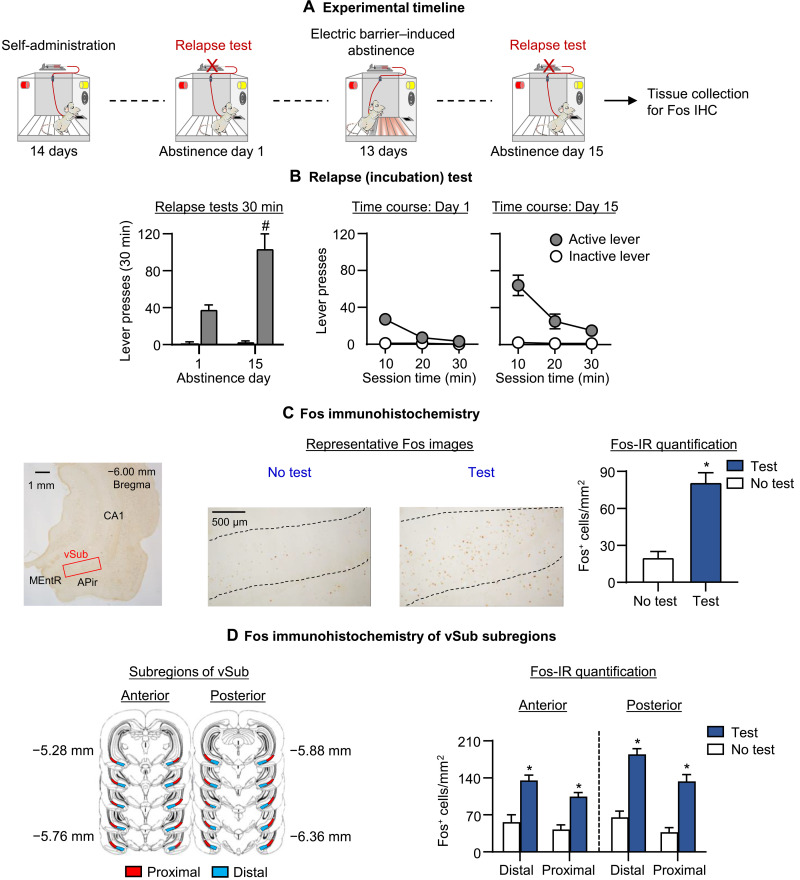
Effect of incubation of oxycodone seeking after electric barrier–induced abstinence on vSub Fos expression. (**A**) Timeline of experiment 1. IHC, immunohistochemistry. (**B**) Relapse (incubation) tests: Means ± SEM number of active lever presses during the 30-min day 1 test session and the first 30-min of the day 15 test session. During testing, active lever presses led to contingent presentation of the tone-light cue previously paired with oxycodone infusions during training, but not oxycodone infusions (extinction conditions). We tested the rats on days 1 and 15 (within-subjects design). (**C**) Fos-positive cells in vSub at ×10 magnification and Fos–immunoreactive (IR) quantification: Number of Fos-positive cells (counts/mm^2^) in rats perfused either immediately after the day 15 test or 24 hours later (no-test and test). (**D**) Fos-IR quantification of anterior-posterior and distal-proximal vSub subregions/areas: Number of Fos-positive cells (counts/mm^2^) in rats perfused either immediately after the day 15 test or 1 day later (no-test and test). * Different from no-test. # Different from day 1, *P* < 0.05. Data are means ± SEM. No test: *n* = 6 (three males, three females); test: *n* = 7 (four males, three females). See fig. S1 for individual data.

Oxycodone seeking in the relapse (incubation) tests was greater after 15 abstinence days than after 1 day, demonstrating incubation of oxycodone craving after electric barrier–induced abstinence (Fig. 2B). The two-way repeated measures (RM)–analysis of covariance (ANCOVA) (inactive lever as a covariate) for the number of active lever presses of rats tested on days 1 and 15, which included the within-subjects factors of abstinence day (1 or 15) and session time (10, 20, or 30 min), showed significant effects of abstinence day (*F*_1,10_ = 6.2, *P* = 0.032), session time (*F*_2,20_ = 17.1, *P* < 0.001), and abstinence day × session time (*F*_2,20_ = 7.0, *P* = 0.005).

#### Fos immunohistochemistry

We measured Fos expression after the day 15 relapse (incubation) test. Relapse to oxycodone seeking during testing was associated with increased Fos expression in vSub (Fig. 2C). The one-way analysis of variance (ANOVA) for Fos-positive cells/mm^2^ in vSub, which included the between-subjects factor of test condition (no-test and test), showed a significant effect of this factor (*F*_1,11_ = 36.3, *P* < 0.001). Representative pictures of Fos expression in the no-test and test rats are shown in Fig. 2C.

#### Fos immunohistochemistry of vSub subregions

We also measured Fos expression after the day 15 relapse (incubation) test in anterior-posterior and distal-proximal vSub subregions (see Fig. 2D, left). Relapse to oxycodone seeking during testing was associated with increased Fos expression in the different vSub subregions with higher relapse-related expression in the posterior than anterior vSub. Within each subregion, Fos expression was higher in the distal than the proximal location. The mixed ANOVA for Fos-positive cells per square millimeter in subregions of vSub, which included the between-subjects factor of test condition and the within-subjects factors of subregion (anterior and posterior), and location (distal and proximal) showed significant effects of test condition (*F*_1,11_ = 318.4, *P* < 0.001), test condition × subregion (*F*_1,11_ = 6.2, *P* < 0.030), and test condition × location (*F*_1,11_ = 7.0, *P* < 0.023).

The results of experiment 1 demonstrate that incubation of oxycodone seeking after electric barrier–induced voluntary abstinence is associated with increased neuronal activity in vSub. This effect was stronger in the posterior than the anterior subregion, and within each subregion, the effect was stronger in the distal than proximal location.

### Global vSub inactivation decreased incubated oxycodone seeking after electric barrier–induced abstinence

In experiment 1, we found that incubation of oxycodone seeking after electric barrier–induced voluntary abstinence was associated with increased Fos expression in vSub. The goal of experiment 2 was to determine a causal role of vSub in this form of incubation using the classical muscimol-baclofen inactivation procedure (*25*). We tested different groups of male and female rats for the effect of vSub saline or muscimol-baclofen injections on oxycodone seeking 1 day after oxycodone self-administration training or after 15 days of electric barrier–induced abstinence.

#### Relapse test

**Fig. 3.**
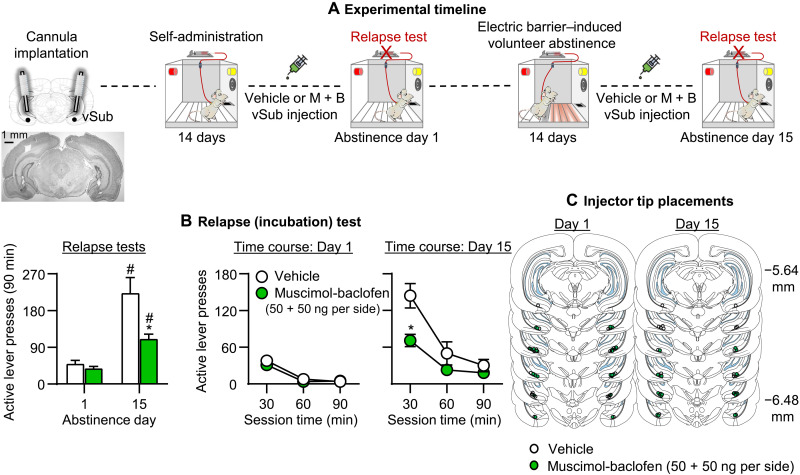
Effect of muscimol-baclofen vSub inactivation on incubation of oxycodone seeking after electric barrier–induced abstinence. (**A**) Timeline of experiment 2. (**B**) Relapse (incubation) tests: Means ± SEM, number of active lever presses during the 90-min test sessions after muscimol-baclofen vSub injection (0 and 50 + 50 ng per side). We tested separate groups of rats on either day 1 or 15 (between-subjects design). (**C**) Injector tip placements in vSub. *Different from vehicle. #Different from day 1, *P* < 0.05. Day 1: *n* = 13 to 14 rats per dose (seven to eight males per dose, five to seven females per dose); day 15: *n* = 10 to 11 rats per dose (five males per dose, five to six females per dose). See fig. S1 for individual data. M + B, muscimol-baclofen.

Muscimol-baclofen vSub inactivation decreased incubated oxycodone seeking on day 15 but had no effect on nonincubated oxycodone seeking on day 1 (Fig. 3B). The mixed factorial ANCOVA (inactive lever as a covariate) for number of active lever presses, which included the between-subjects factors of abstinence day (1 to 15) and muscimol-baclofen dose (0 and 50 + 50 ng per side), and the within-subjects factor of Session time (30, 60, or 90 min) showed significant effects of abstinence day (*F*_1,43_ = 43.3, *P* < 0.001), muscimol-baclofen dose (*F*_1,43_ = 10.5, *P* = 0.002), session time (*F*_2,86_ = 62.7, *P* < 0.001), and abstinence day × muscimol-baclofen dose (*F*_11,43_ = 7.3, *P* = 0.01). The results of experiment 2 demonstrate that global inhibition of vSub neuronal activity selectively decreased incubated, but not nonincubated, oxycodone seeking.

### Global vSub inactivation had no effect on incubated oxycodone seeking after forced abstinence

The goal of experiment 3 was to determine whether the effect of muscimol-baclofen vSub inactivation on incubated oxycodone seeking after electric barrier–induced voluntary abstinence would generalize to incubation after forced abstinence. For this purpose, we tested different groups of rats for the effect of saline or muscimol-baclofen injections into the vSub on oxycodone seeking after 15 days of forced abstinence from oxycodone self-administration.

#### Relapse test

**Fig. 4.**
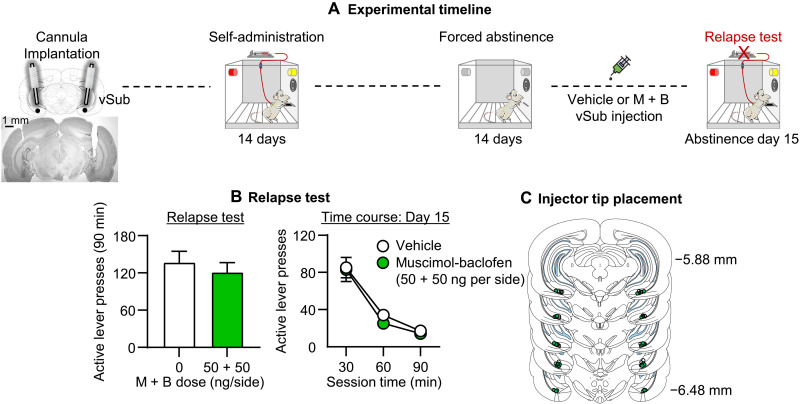
Effect of muscimol-baclofen vSub inactivation on incubation of oxycodone seeking after forced abstinence. (**A**) Timeline of experiment 3. (**B**) Relapse (incubation) test: Means ± SEM number of active lever presses during the 90-min day 15 test session after muscimol-baclofen vSub injection (0 and 50 + 50 ng per side). (**C**) Injector tip placements in vSub. *n* = 16 to 18 rats per dose (8 to 10 males per dose, 8 females per dose). See fig. S1 for individual data. M + B, muscimol-baclofen.

Inactivation of the vSub with muscimol-baclofen had no effect on incubated oxycodone seeking on day 15 after forced abstinence (Fig. 4B). The mixed factorial ANCOVA (inactive lever as a covariate) for number of active lever presses of rats on day 15, which included the between-subjects factor of muscimol-baclofen dose (0 and 50 + 50 ng per side) and the within-subjects factor of session time (30, 60, and 90 min), showed a significant effect of session time (*F*_2,62_ = 30.2, *P*< 0.001) but no significant effects of muscimol-baclofen dose or interaction (*P* values of >0.1). The results of experiment 3 demonstrate that global inhibition of vSub neuronal activity had no effect on incubation of oxycodone seeking after forced abstinence.

### Selective inactivation of vSub neuronal ensembles decreased incubated oxycodone seeking after electric barrier–induced abstinence

In experiment 2, we found that muscimol-baclofen inactivation of vSub decreased incubated oxycodone seeking after electric barrier–induced abstinence. These data indicate that vSub activity contributes to the incubated oxycodone seeking. However, from the perspective of the putative role of vSub-activated neuronal ensembles and their role in incubation, the interpretation of the muscimol-baclofen data is confounded by the fact that this manipulation inhibits all neurons in a given brain area, independent of their task-related activity (incubated oxycodone seeking in our study). Therefore, the goal of experiment 4 was to determine whether selective inactivation of oxycodone relapse-associated vSub neuronal ensembles would decrease incubation of oxycodone seeking after electric barrier–induced abstinence.

We trained male and female *Fos-LacZ* transgenic rats for 14 days of oxycodone self-administration and 14 days of electric barrier–induced abstinence. One day later (abstinence day 15), we exposed different groups of rats to either a 15-min “induction” session under extinction conditions in the self-administration chambers, during which lever presses were reinforced by the oxycodone-paired cues but not oxycodone (to activate the putative relapse-associated neuronal ensembles), or 15 min in a novel context as a control that activates neuronal ensembles independent of the relapse-associated ensembles (to induce relapse-independent Fos expression). Ninety minutes after the induction session, we injected the rats with either saline or Daun02 (4 μg per side) into the vSub to inactivate the relapse-associated and relapse-independent Fos-positive neurons and tested all rats 3 days later (abstinence day 18) for incubated oxycodone seeking.

#### Induction day (day 15)

On induction day, active lever pressing in the 15-min oxycodone seeking session was not statistically different between the groups that received vehicle or Daun02 injections 90 min after the session’s onset (means ± SEM active lever presses per 15 min: vehicle, 91 ± 18; Daun02, 81 ± 10; *F*_1,23_ = 0.02, *P* = 0.90).

#### Relapse test (day 18)

**Fig. 5.**
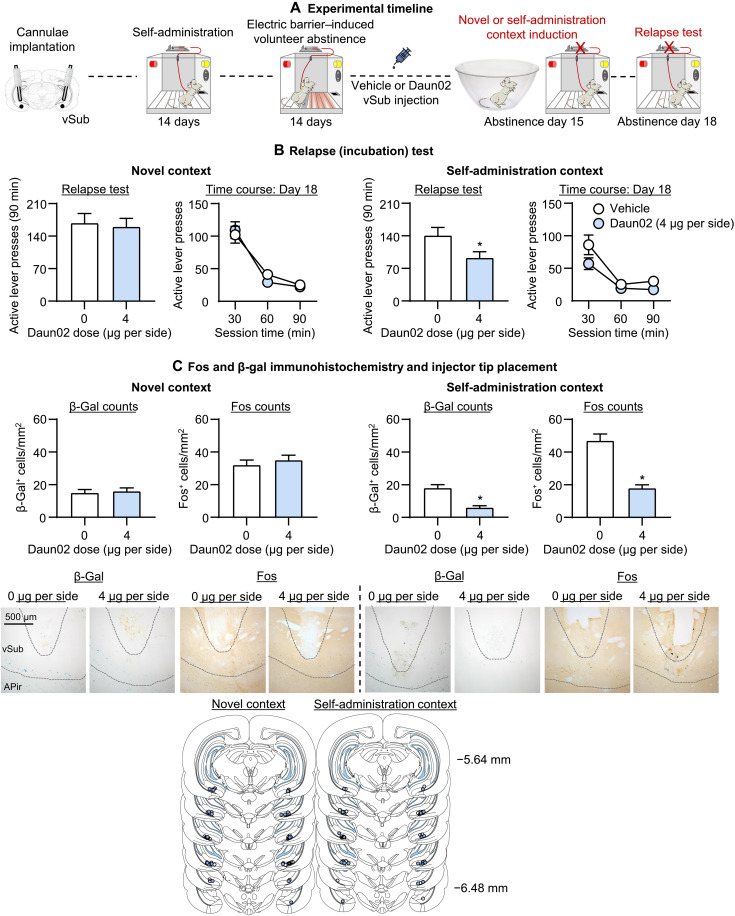
Effect of Daun02 vSub inactivation on incubation of oxycodone seeking after electric barrier–induced abstinence. (**A**) Timeline of experiment 4. (**B**) Relapse (incubation) tests: Means ± SEM number of active lever presses during the day 18 90-min test session after Daun02 vSub injection (0 and 4 μg per side) on day 15. (**C**) Representative images of β-gal^+^ and Fos^+^ cells in vSub at ×10 magnification; β-gal and Fos-IR quantification: means ± SEM number of β-gal-positive or Fos-positive cells (counts/mm^2^) in rats perfused after the day 18 relapse test and injector tip placements in vSub. *Different from vehicle, *P* < 0.05. Novel context: *n* = 16 to 17 rats per dose (6 to 9 males per dose, 7 to 11 females per dose); self-administration context: *n* = 12 to 14 rats per dose (6 to 9 males per dose, 7 to 11 females per dose). See fig. S1 for individual data.

We analyzed the data separately for saline versus Daun02 for the two induction conditions (drug-seeking session in the self-administration chambers versus novel context), because their experimental conditions before the relapse test were different (no oxycodone seeking under extinction conditions for the novel context group). Daun02 inactivation of vSub neurons activated during the short 15-min oxycodone-seeking session (abstinence day 15) decreased oxycodone seeking 3 days later (Fig. 5B). The mixed factorial ANCOVA (inactive lever as a covariate) for the number of active lever presses, which included the between-subjects factor of Daun02 dose (0 and 4 μg per side) and the within-subjects factor of session time (30. 60, 90 min), showed significant effects of Daun02 dose (*F*_1,23_ = 4.5, *P* = 0.044) and session time (*F*_2,46_ = 20.9, *P* < 0.001) but no significant interaction (*P* > 0.1). In contrast, Daun02 inactivation of vSub neurons activated during a short 15-min novel context session (abstinence day 15) had no effect on oxycodone seeking 3 days later. The ANCOVA showed a significant effect of session time (*F*_2,60_ = 39.1, *P* < 0.01) but not Daun02 dose or an interaction (*P* values of >0.1).

#### *Fos and* β*-gal histochemistry*

On the day 18 relapse test session, prior Daun02 injections decreased the number of both Fos and β-gal–labeled neurons in vSub of rats exposed to the short oxycodone-seeking session under extinction conditions on induction day, but not in rats exposed to the novel context (Fig. 5C). These results indicate that the Daun02 manipulation selectively inactivated relapse-associated vSub neurons. One-way ANOVAs for Fos-positive cells per square millimeter and β-gal-positive cells per square millimeter in vSub, which included the between-subjects factor of Daun02 dose (0 and 4 μg per side), showed a significant effect of this factor for Fos (*F*_1,24_ = 50.3, *P* < 0.001) and β-gal (*F*_1,24_ = 33.1, *P* < 0.001). In contrast, Daun02 inactivation of novel context-associated neurons in the vSub on induction day did not decrease Fos and β-gal–labeled neurons in vSub after the 90-min relapse test (*P* values of >0.1). Representative pictures of Fos and β-gal staining are shown in Fig. 5C.

We also determined Fos and β-gal double labeling in vSub of two rats exposed to a novel context for 90 min to verify that our Daun02 manipulation targets behaviorally activated Fos-expressing neurons. We found that 75 ± 3% of β-gal are colocalized with Fos in vSub (fig. S2). This percentage of coexpression (double labeling) is similar to those we observed in our previous studies in other brain areas (*31*–*33*, *44*). The lower than 100% coexpression likely reflects differences in the sensitivity of our assay conditions for detecting Fos versus β-gal. The results of experiment 4 demonstrate a critical role of neuronal ensembles in the vSub for incubated oxycodone craving after electric barrier–induced voluntary abstinence.

### Molecular phenotyping of vSub Fos-positive neurons with FACS + qPCR

In experiment 4, we found that selective inactivation of vSub Fos-expressing cells decreased oxycodone seeking, suggesting a role of Fos-expressing neuronal ensembles in incubated oxycodone seeking after electric barrier–induced voluntary abstinence. In experiment 5, we determined that the molecular phenotypes of the relapse-associated vSub activated Fos-positive neurons. We tested male and female rats for oxycodone seeking under extinction conditions after 15 days of electric barrier–induced abstinence. Immediately after the 90-min relapse test on abstinence day 15, we euthanized the rats and extracted their brains for FACS processing of vSub tissue and subsequent qPCR of selected constitutive genes (see table S2). The selected genes are based, in part, on our previous FACS studies (*39*–*41*).

#### Relapse test

During the day 15 relapse test, lever presses on the active lever were higher than on the inactive lever (fig. S4C). The one-way RM-ANOVA for number of lever presses, which included the within-subjects factors of lever (active and inactive) and session time (30, 60, or 90 min), showed a significant effect of lever × session time (*F*_2,24_ = 36.5, *P* < 0.001).

#### Fluorescence-activated cell sorting

We isolated Fos-positive and Fos-negative vSub neurons by labeling the neurons with NeuN and Fos antibodies and sorted them using our previously described FACS procedure (*38*–*40*, *45*). We first identified cells from debris based on the distinct forward and side scatter properties (fig. S4D, left). Then, within the gate for single cells, we identified all neurons immunolabeled with NeuN antibody (fig. S4D, middle) and activated neurons immunolabeled with both NeuN and Fos antibodies (upper gate in fig. S4D, right). Last, we sorted activated neurons (Fos-positive/NeuN-positive) from nonactivated neurons (Fos-negative/NeuN-positive) (see fig. S4D, right, for a representative example from one sample).

#### Quantitative polymerase chain reaction

We used qPCR to assess expression of molecular markers associated with different neurotransmission (both via neurotransmitters or neuropeptides) in the Fos-positive and Fos-negative neurons. In both Fos-positive and Fos-negative neurons, we detected expression of the acetylcholine receptor–related genes *Chrna2-7 and Chrm1-5*; the dopamine receptor-related genes *Drd1 and Drd2*; the GABA-related genes *Gabra1*, *Gabra3*, *Gabra5*, *Gabbr2*, and *Gabbrg2*; the glutamate-related genes *Gria1-4*, *Grin1*, *Grin2a*,*b*, *Grm1-5*; and the opioid-related genes *Oprm1*, *Oprd1*, and *Opcml* (table S2). The complete analyses of the gene expression within the Fos-positive and Fos-negative neuron populations are described in table S2. These results indicate that the relapse-activated vSub neuronal ensembles (the Fos-positive neurons) are composed of cells expressing cholinergic, dopaminergic, glutamatergic, GABAergic, and opioid-related receptor genes.

### Longitudinal functional connectivity changes in vSub-related circuits predict incubation

**Fig. 6.**
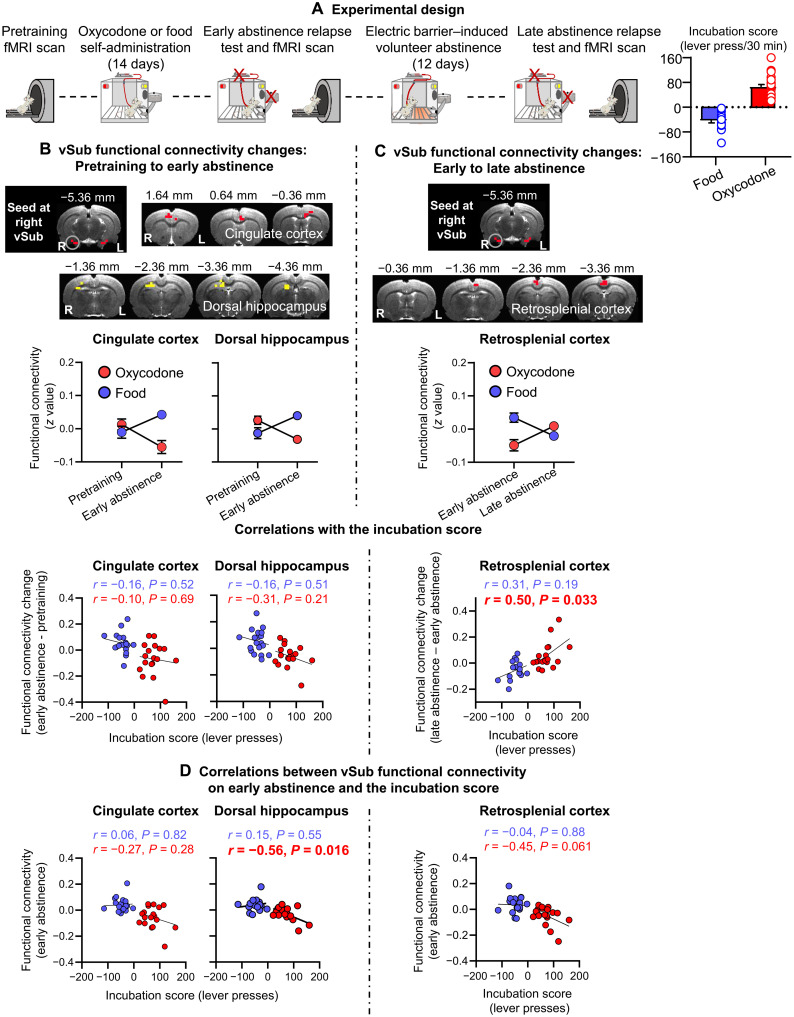
vSub functional connectivity: Initial analysis with a seed at the right vSub. (**A**) Experimental timeline and the incubation score. (**B**) Self-administration phase: Significant reward (food, oxycodone) × time (pretraining, early abstinence) interaction in functional connectivity of vSub with aCC and dHipp (interaction regions and interaction patterns) and correlations between the changes in the vSub-aCC and vSub-dHipp connectivity and the incubation score. (**C**) Voluntary abstinence phase: Significant reward (food, oxycodone) × time (early abstinence, late abstinence) interaction in functional connectivity of vSub with retrosplenial cortex (interaction regions and interaction patterns) and correlation between the change in the vSub-RSC connectivity and the incubation score. (**D**) Early abstinence (day 2): Correlations between vSub functional connectivity at early abstinence and the incubation score. Imaging results were corrected for whole-brain multiple comparisons at *P*_corr_ < 0.025. Data are means ± SEM. Food group: *n* = 19; oxycodone group: *n* = 18. The underlay brain images in (B) and (C) are from a rat brain MRI template that was registered to a rat brain atlas (*80*). The incubation score graph is from Fredriksson *et al.* (*18*).

We conducted whole-brain voxel-wise functional connectivity analyses with the right or left vSub (Fig. 6B) as a seed to determine whether longitudinal vSub-related functional connectivity changes induced by either oxycodone (or food) self-administration (pretraining to early abstinence; Fig. 6A) or voluntary abstinence (early abstinence to late abstinence; Fig. 6A) would predict incubation of oxycodone (or abatement of food) seeking. We also determined whether functional connectivity at early abstinence would predict incubation.

### Initial analyses: vSub seed

#### Self-administration phase and early abstinence

The ANOVA with the right vSub as a seed showed a significant reward (food, oxycodone) × time (pretraining, early abstinence) interaction in connectivity of the right vSub with anterior cingulate cortex (aCC) and dorsal hippocampus (dHipp) (Fig. 6B). Post hoc analyses showed that the interaction is due to decreased vSub-aCC and vSub-dHipp connectivity in the oxycodone group and increased connectivity in the food group. The change in vSub-aCC or vSub-dHipp connectivity was not correlated with the incubation score (Fig. 6B). However, vSub-dHipp connectivity at early abstinence was negatively correlated with the incubation score in the oxycodone group but not the food group (Fig. 6D). The ANOVA with the left vSub as a seed did not show any significant interactions.

#### Voluntary abstinence phase

The ANOVA with the right vSub as a seed showed a significant reward × time (early abstinence, late abstinence) interaction in connectivity of the right vSub with retrosplenial cortex (RSC) (Fig. 6C). Post hoc analyses showed that the interaction was due to increased vSub-RSC connectivity in the oxycodone group and decreased connectivity in the food group. The change in the vSub-RSC connectivity was positively correlated with the incubation scores in the oxycodone group but not the food group. vSub-RSC connectivity at early abstinence was marginally (corrected *P* value of 0.061) negatively correlated with the incubation score in the oxycodone group but not the food group (Fig. 6D). The ANOVA with the left vSub as a seed did not show any significant interactions.

Together, the initial fMRI analyses showed that voluntary abstinence-related functional connectivity changes (early to late abstinence) of the right (but not left) vSub with RSC predicted incubation of oxycodone (but not food) seeking. In addition, the early abstinence functional connectivity of the right vSub with dHipp predicted this incubation.

### Follow-up analyses: dHipp and RSC

For brain regions (dHipp and RSC) showing significant interactions and correlations with the incubation score in the above first-level ANOVAs, we conducted follow-up analyses to determine whether functional connectivity changes in these regions induced by self-administration or voluntary abstinence would predict incubation of oxycodone (or food) seeking.

#### dHipp seed

**Fig. 7.**
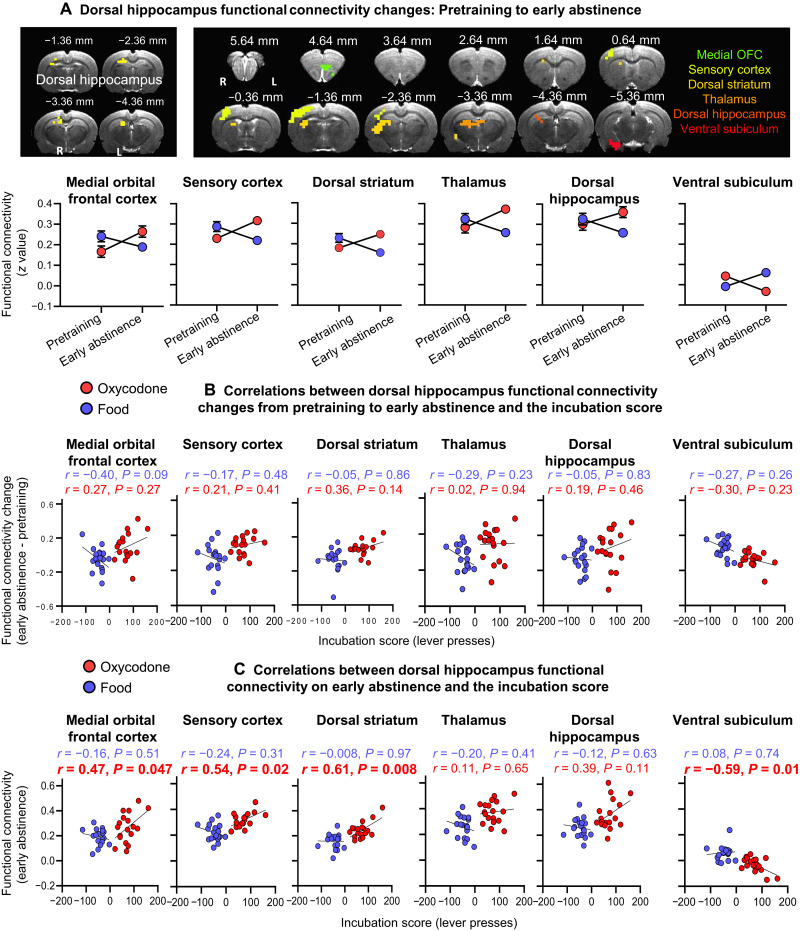
vSub functional connectivity changes: Follow-up analyses with a seed in the dHipp. (**A**) Self-administration phase: Significant reward × time (pretraining, early abstinence) interactions in functional connectivity of dHipp with medial OFC, sensory cortex, dorsal striatum, thalamus, dHipp, and vSub (interaction regions and interaction patterns) and (**B**) correlations between the changes in the dHipp functional connectivity and the incubation score. (**C**) Early abstinence (day 2): Correlations between dHipp functional connectivity at early abstinence and the incubation score. Imaging results were corrected for whole-brain multiple comparisons at *P*_corr_ < 0.025. Data are means ± SEM. Food group: *n* = 19; oxycodone group: *n* = 18. The underlay brain images in (A) are from a rat brain MRI template that was registered to a rat brain atlas (*80*).

The ANOVAs showed significant reward (food and oxycodone) × time (pretraining and early abstinence) interactions in connectivity of the dHipp with medial OFC, sensory cortex, dorsal striatum, thalamus, dHipp, and vSub. (Fig. 7A). Post hoc analyses showed that the interactions were due to increased connectivity of dHipp with the above regions (except the vSub) in the oxycodone group and decreased connectivity in the food group. For vSub, the significant interaction was due to decreased dHipp-vSub connectivity in the oxycodone group and increased connectivity in the food group. The dHipp connectivity changes during the self-administration phase were not correlated with the incubation score (Fig. 7B). In contrast, dHipp connectivity with medial OFC, sensory cortex, and dorsal striatum at early abstinence was positively correlated with the incubation score in the oxycodone group but not the food group (Fig. 7C). In contrast, the dHipp-vSub connectivity at early abstinence was negatively correlated with the incubation score in the oxycodone group but not the food group (Fig. 7C).

#### Retrosplenial cortex

The ANOVA with the RSC as a seed did not show any significant interactions during the voluntary abstinence phase. Together, the follow-up analyses with dHipp as the seed showed that dHipp connectivity with medial OFC, sensory cortex, dorsal striatum, and vSub at early abstinence predicted incubation of opioid seeking. In contrast, follow-up analysis with RSC as the seed did not identify any functionally connected brain areas predictive of this incubation.

## DISCUSSION

We studied the role of vSub in incubation of opioid craving after voluntary abstinence induced by adverse consequences of drug seeking. We found that incubated oxycodone seeking was associated with increased vSub Fos expression. In addition, global inactivation of vSub with muscimol-baclofen decreased incubated (day 15), but not nonincubated (day 1), oxycodone seeking. In contrast, muscimol-baclofen inactivation of vSub had no effect on incubation after forced abstinence. Moreover, Daun02 inactivation of vSub neurons previously activated by exposure to the oxycodone self-administration context and discrete cues during a short “memory reactivation” induction session decreased incubated oxycodone seeking and vSub activity 3 days later. In contrast, Daun02 inactivation of a distinct set of vSub neurons previously activated by novel context exposure had no effect. The FACS and qPCR analysis of the relapse-activated vSub neuronal ensembles (the Fos-positive neurons) showed that they are composed of cells expressing cholinergic, dopaminergic, glutamatergic, GABAergic, and opioid-related receptor genes. Together, these results indicate a selective role of vSub neuronal ensemble activity in incubation of oxycodone seeking after voluntary, but not forced, abstinence.

Last, exploratory analysis of brain images from Fredriksson *et al.* (*18*) showed that voluntary abstinence-related functional connectivity changes between vSub and RSC and early abstinence-related functional connectivity changes between vSub and dHipp predicted incubation of oxycodone seeking. Follow-up analyses showed that early abstinence-related functional connectivity changes between dHipp and medial OFC, sensory cortex, dorsal striatum, and vSub also predicted this incubation.

### Role of vSub in incubation of opioid craving after voluntary, but not forced, abstinence

Initial studies on vSub (and other ventral hippocampus areas) role in drug relapse/reinstatement focused on psychostimulants. Electrical stimulation of vSub reinstates cocaine or amphetamine seeking (*46*, *47*). Muscimol-baclofen or lidocaine inactivation of ventral hippocampus or vSub decreases cue- and cocaine-induced reinstatement of cocaine and methamphetamine seeking (*48*–*50*). Muscimol-baclofen inactivation of ventral hippocampus decreases context-induced reinstatement of cocaine seeking (*51*). Previously, we showed a role of vSub and vSub→nucleus accumbens shell projections in context-induced reinstatement of heroin seeking after extinction (*20*, *21*) and context-induced relapse to alcohol seeking after punishment-induced abstinence (*19*). Together, these studies indicate a general role of ventral hippocampus in relapse across drug classes and relapse models. However, our data do not support this notion. We found a selective role of vSub in incubation of oxycodone seeking after electric barrier–induced abstinence, but not incubation, after forced abstinence or “nonincubated” oxycodone seeking during early abstinence (day 1).

The different effects of vSub inactivation on incubation of oxycodone seeking after electric barrier–induced abstinence versus forced abstinence are likely due to the different methods used to achieve abstinence (*52*, *53*). For example, basolateral amygdala activity plays opposite roles in context-induced relapse of cocaine seeking after extinction versus punishment (*54*). In addition, central amygdala protein kinase δ and somatostatin play dissociable roles in incubation of methamphetamine craving after forced abstinence versus prevention of this incubation after voluntary abstinence induced by rewarding social interaction (*55*).

### Role of vSub neuronal ensembles in incubation of opioid craving

We used the Daun02 inactivation method (*26*) to study the causal role of vSub neuronal ensembles in incubation of opioid craving after electric barrier–induced abstinence. This method has been used to selectively inactivate behaviorally activated (Fos-positive) neurons (*27*). In this regard, muscimol-baclofen and other reversible inactivation methods, site-specific receptor antagonist/agonist injections, or cell type–specific optogenetic and chemogenetic methods are not suitable to study neuronal ensembles’ role in learned behaviors (*27*). This is because these methods invariably inhibit both behaviorally activated and nonactivated neurons.

vSub Daun02 inactivation decreased incubated oxycodone seeking after electric barrier–induced abstinence, demonstrating a role of vSub neuronal ensembles in this incubation. In addition, we used FACS and qPCR to begin to identify the molecular phenotype of vSub Fos-expressing neuronal ensembles. We detected several constitutive genes in these ensembles (table S2), indicating that they are composed of cells expressing cholinergic, dopaminergic, glutamatergic, GABAergic, and opioid-related receptor genes. A question for future studies is which specific vSub cell types are causally involved in incubation of oxycodone craving. We speculate that the putative vSub incubation-related ensembles are not cell type specific. In previous relapse/reinstatement and incubation studies, we found no evidence for cell type specificity (Drd1-expressing versus Drd2-expressing striatal cells or GABA-expressing versus glutamate-expressing medial prefrontal cortex cells) of Fos-expressing neuronal ensembles (*33*, *56*, *57*).

### Longitudinal functional connectivity changes in vSub-related circuits predict incubation

We recently reported that longitudinal resting-state functional connectivity changes in OFC with dorsal striatum and related circuits predict incubation of oxycodone opioid craving after electric barrier–induced abstinence (*18*). Here, we used the brain images from this study, which also included a nondrug food self-administration group, to begin to investigate vSub-related circuits that potentially contribute to this incubation.

The main findings from the initial analyses with vSub as a seed were that voluntary abstinence-induced functional connectivity changes (from early to late abstinence; Fig. 6A) of vSub with RSC predicted (positive correlation) the incubation score (Fig. 6C) and that the early abstinence (day 2) functional connectivity of vSub with dHipp predicted (negative correlation) this score (Fig. 6D). Early abstinence functional connectivity of vSub with RSC also appeared to predict the incubation score (negative correlation), but this effect did not reach formal statistical significance (*P* = 0.061; Fig. 6D). The main findings from the follow-up analyses with dHipp as a seed were that the early abstinence (day 2) functional connectivity of dHipp with medial OFC, sensory cortex, dorsal striatum, and vSub predicted the incubation score with positive correlations for medial OFC, sensory cortex, and dorsal striatum and negative correlation for vSub (Fig. 7D).

These exploratory fMRI analyses are correlational and will be instrumental in identifying brain areas and projections for future investigation of the role of vSub-related circuits in incubation of opioid craving. In this regard, the connectivity analyses identified brain regions such as dHipp and dorsal striatum previously implicated in relapse-related behaviors after extinction or forced abstinence in rat models (*58*, *59*), including incubation of drug craving after forced abstinence or food choice–induced voluntary abstinence (*33*, *39*, *60*).

Perhaps, more importantly, our exploratory analyses also identified areas such as the sensory cortex and RSC that were not previously implicated in relapse-related behaviors in animal models. The RSC is of particular interest because it is a primary hub of the rat’s default mode network (*42*), analogous to posterior cingulate cortex (pCC) in the human’s default mode network (*61*). Increased activity in pCC and other components of this network is associated with cue-induced cocaine craving in humans (*62*). In addition, we (the Yang laboratory) previously reported that the functional connectivity of Hipp-pCC circuit (closely related to the vSub-RSC circuit in rats) in people who use cocaine predicts relapse (*63*).

### Methodological considerations

One potential issue in our study is that the effects of muscimol-baclofen or Daun02 on lever presses are due to nonspecific performance deficits. This is unlikely because muscimol-baclofen injections had no effect on incubation after forced abstinence, and Daun02 injections after novel context exposure had no effect on incubation after electric barrier–induced abstinence. Another issue is that Daun02 injections decreased incubated oxycodone seeking by nonspecifically inactivating a random group of Fos-positive neurons. This is unlikely because vSub Daun02 injections after novel context exposure, a manipulation that increases Fos expression in vSub (*64*) and other cortical areas (*65*), had no effect on incubation.

Another issue is that the magnitude of the inhibitory effect of Daun02 inactivation on incubated oxycodone seeking (~35%; Fig. 5B) was weaker than that of muscimol-baclofen (~50%; Fig. 3B). In this regard, Daun02 inactivation only partially interferes with the function of incubation-related neuronal ensembles because in vSub and other areas (*31*, *33*, *44*), β-gal is expressed in most (~75%; see Results and fig. S2), but not all, Fos-expressing neurons.

A final issue is that the number of Fos-positive neurons after the relapse test in the vehicle condition of the Daun02 experiment (Fig. 5C) was lower than the number of Fos-positive neurons under similar test conditions in experiment 1 (Fig. 2C). Typically, there is a lower number of Fos-expressing neurons in intact brain tissues versus brain tissues collected after intracranial drug injections due to tissue damage (*33*, *66*). In the same experiment, the number of β-gal–positive neurons was lower than the Fos-positive neurons [Fig. 5C; for similar results, see (*26*, *33*, *57*)]. This difference is likely due to differences in sensitivity of the X-gal assay (an enzymatic assay that does not depend on antibody binding to antigen) versus Fos immunohistochemistry.

In conclusion, we recently developed a rat model of incubation of craving after electric barrier–induced voluntary abstinence (*7*, *18*) and proposed that our model mimics, to some degree, human voluntary abstinence due to negative consequences of drug seeking before drug taking (*16*). Here, we used the activity marker Fos and pharmacological and chemogenetic methods to demonstrate that vSub neuronal ensembles contribute to incubation of opioid craving after abstinence induced by negative consequences of drug seeking. Next, we showed that vSub activity did not contribute to incubation of opioid craving after forced abstinence, suggesting distinct mechanisms of incubation after forced versus voluntary abstinence. We also showed that voluntary abstinence-related functional connectivity of vSub with RSC, as well as early-abstinence functional connectivity of vSub with dHipp, and dHipp with medial OFC and other areas, predicted incubated opioid seeking. Last, it is important to point out that opioids are not a homogenous drug class. Even opioids with high affinity to the mu opioid receptor significantly differ in terms of pharmacodynamics (*67*) and in their ability to increase dopaminergic transmission (*68*, *69*). Thus, a question for future research is whether the role of vSub neuronal ensembles on incubated oxycodone seeking generalizes to heroin and other opioid drugs.

## MATERIALS AND METHODS

### Subjects

In experiments 1 to 3 and 5, we used male (*n* = 74) and female (*n* = 62) Sprague-Dawley rats (Charles River Laboratories), weighing 270 to 390 g and 180 to 240 g, respectively, before surgery. In experiment 4, we used male (*n* = 40) and female (*n* = 46) *Fos-lacZ* transgenic rats (*26*), weighing 300 to 530 g and 180 to 320 g, respectively, before surgery. We maintained the rats under a reverse 12-hour light:12-hour dark cycle (8:00 a.m., lights off) with food and water freely available. We housed two rats per cage before surgery and then individually after surgery. We performed the experiments in accordance with the National Institutes of Health (NIH) *Guide for the Care and Use of Laboratory Animals* (8th edition), under a protocol approved by the local Animal Care and Use Committee. We excluded 55 of the 209 rats used in the study due to catheter failure (*n* = 1), failure to acquire oxycodone self-administration (*n* = 2), poor health (*n* = 19), cannula misplacement (*n* = 18), loose head caps/damaged brains (*n* = 12), reaction to intracranial injection (*n* = 1), or outliers during the relapse tests, >3 SDs from the mean (*n* = 2).

### Drugs

We received oxycodone hydrochloride (HCl) from National Institute on Drugs and Addiction pharmacy and dissolved it in sterile saline. We chose a unit dose of 0.1 mg/kg for self-administration training based on our previous studies (*7*, *70*). In experiments 2 and 3, we dissolved muscimol-baclofen (Tocris Bioscience) in sterile saline and injected it intracranially at a dose of 50 + 50 ng in 0.5 μl per side (*66*, *71*, *72*) 15 to 30 min before the relapse test sessions. In experiment 4, we dissolved Daun02 (Sequoia Research Products) in vehicle solution containing 5% dimethyl sulfoxide, 6% Tween-80, and 89% 0.1 M sterile phosphate-buffered saline (PBS) and injected it intracranially at a dose of 4 μg in 1.0 μl per side 90 min after the induction session. We chose the Daun02 concentration based on our previous study (*33*).

### Intravenous surgery

We anesthetized the rats with isoflurane (5% induction, 2 to 3% maintenance, Covetrus). We attached silastic catheters to a modified 22-gauge cannula cemented to polypropylene mesh (Amazon or Industrial Netting), inserted the catheter into the jugular vein, and fixed the mesh to the mid-scapular region of the rat (*33*, *73*, *74*). We injected the rats with ketoprofen (2.5 mg/kg, s.c.; Covetrus) after surgery, and the following day, to relieve pain and decrease inflammation. We allowed the rats to recover for 6 to 8 days before oxycodone self-administration training. During recovery and all experimental phases, we flushed the catheters every 24 to 48 hours with gentamicin (4.25 mg/ml; Fresenius Kabi, USA) dissolved in sterile saline. If we suspected catheter failure during training, we tested patency with the short-acting barbiturate anesthetic Brevital [methohexital sodium, Covetrus; 10 mg/ml in sterile saline, 0.1- to 0.2-ml injection volume (i.v.)], and if not patent, we recatheterized the other jugular vein and continued training the next day or eliminated the rat from the study.

### Intracranial surgery

We performed the intracranial surgery at the same time of the intravenous surgery for all experiments except for the rats in experiment 2 in which we tested for relapse on abstinence day 15. For these rats, we performed the intracranial surgery 1 day after the last day of oxycodone self-administration training. We gave the rats 3 days to recover before the voluntary abstinence (electric barrier) phase (see below). We anesthetized the rats and, using a stereotaxic instrument (Kopf), implanted bilateral guide cannulas (23-gauge; Plastics One) 1 mm above the vSub. We set the nose bar at −3.3 mm and used the following coordinates from bregma: anteroposterior, −6.0 mm; mediolateral, ±5.3 mm (4° angle); dorsoventral, −7.5 mm for males and −7.2 to 7.5 mm for females. We anchored the cannulas to the skull with jeweler’s screws and dental cement. We used the above coordinates based on our previous studies (*19*–*21*).

### Intracranial injections

Four days before the intracranial injections, we habituated the rats to the injection procedure. Habituation consisted of three phases. We first exposed the rats to the injection cage (an empty cage containing bedding). The following day, we gently removed the cannula blockers before exposing the rats to the injection cage. On the last day of habituation, we gently lowered down the injectors and placed them in the injection cage. On the test day, we connected the syringe pump (Harvard Apparatus) to 10-μl Hamilton syringes and attached the Hamilton syringes to the 30-gauge injectors via polyethylene-50 tubing; the injectors were extended 1 mm below the tips of the guide cannulas. In experiment 2 to 3, we injected vehicle (saline) or muscimol-baclofen (50 + 50 ng in 0.5 μl per side) at a rate of 0.5 μl/min and left the injector in place for an additional minute to allow diffusion. After testing, we deeply anesthetized the rats with isoflurane and removed their brains and stored them in 10% formalin. We sectioned brains at 50 μm using a Leica Microsystems cryostat and stained sections with cresyl violet to verify the placement of the cannulas. In experiment 4, we injected vehicle or Daun02 (4 μg/1.0 μl per side) at a rate of 0.5 μl/min and left the injectors in place for two additional minutes to allow diffusion.

### Fos immunohistochemistry

We based our Fos immunohistochemistry procedure on our previous reports (*20*, *75*). Ninety minutes after the relapse test in experiments 1 and 4, we deeply anesthetized the rats with isoflurane saturated in air in an enclosed glass desiccator for 80 s and perfused them transcardially with 100 ml of 1× PBS (pH 7.4) followed by ∼400 ml of 4% paraformaldehyde in PBS. In experiment 1, we also perfused the no-test rats (taken from their homecage) the day following the relapse test. We removed and postfixed the brains in 4% paraformaldehyde for 2 hours before transferring them to 30% sucrose in PBS for 48 hours at 4°C. We subsequently froze the brains in powdered dry ice and stored them at −80°C until sectioning. We cut coronal sections (40 μm) containing the vSub using a cryostat (Leica Microsystems). We divided the sections into five series (200 μm apart) and stored them in PBS containing 0.1% sodium azide at 4°C.

We rinsed free-floating sections in PBS (3×, 10 min), incubated them for 1 hour in 4% bovine serum albumin (BSA) in PBS with 0.3% Triton X-100 (PBS-TX), and incubated them overnight at 4°C with rabbit anti–c-Fos primary antibody [phospho–c-Fos (Ser^32^), Cell Signaling Technology, RRID: AB_2247211; D82C12 diluted 1:8000] in 4% BSA in 0.3% PBS-TX. We then rinsed the sections in PBS and incubated them for 2 hours with biotinylated anti-rabbit immunoglonbulin G secondary antibody (BA-1000, Vector Laboratories) diluted 1:600 in 4% BSA in 0.4% PBS-TX. We rinsed the sections again in PBS and incubated them in avidin-biotin-peroxidase complex (the ABC Elite Kit, PK-6100, Vector Laboratories) in 0.5% PBS-TX for 1 hour. We then rinsed the sections in PBS, developed them in 3,3′-diaminobenzidine, rinsed them in PBS, mounted them onto chrome alum/gelatin-coated slides, and air-dried them.

We dehydrated the slides through a graded series of alcohol concentrations (30, 60, 90, 95, and 2 × 100% ethanol), cleared with Citra Solv (Thermo Fisher Scientific), and coverslipped them with Permount (Thermo Fisher Scientific). We captured bright-field images of vSub with a Retiga 2000R charge-coupled device (CCD) camera (QImaging) attached to a Zeiss microscope Axio Scope A1 using a 10× objective. We counted Fos–immunoreactive (IR) nuclei, characterized by brown nuclear staining, using iVision (4.5.0, Biovision Technologies). For each rat, we quantified cells in both hemispheres of two sections and computed a mean of these counts per area. We first captured images and quantified cells around the Bregma coordinate: −6.0 (Fig. 2C). Next, we divided vSub into distal and proximal locations (areas) and captured images and quantified cells at two different Bregma coordinates: −5.3 to −5.8 and −5.9 to −6.3. We defined the former anterior vSub and the latter posterior vSub (see Fig. 2D for the selected subregions of vSub). Two independent blind observers performed image-based quantification of Fos-labeled neurons [interrater reliability for counting between Ida Fredriksson and Carlo Cifani (*r* = 0.99, *P* < 0.01) and Ida Fredrkisson and Ashley Batista (*r* = 0.99, *P* < 0.01) for experiment 1 and Ida Fredriksson and Aniruddha Shekara (*r* = 0.99, *P* < 0.01) for experiment 4].

### X-gal histochemistry for β-gal visualization in *Fos-lacZ* rats

The X-gal assay is based on our previous studies (*26*, *31*, *57*). Ninety minutes after the relapse tests in experiment 4, we deeply anesthetized the rats with isoflurane saturated in air in an enclosed glass desiccator for 80 s and perfused them transcardially with ∼100 ml of 0.1 m of PBS (pH 7.4), followed by ∼400 ml of 4% paraformaldehyde in PBS. We removed the brains and postfixed them in 4% paraformaldehyde for 2 hours before transferring them to 30% sucrose in PBS for 48 hours at 4°C. We froze the brains in dry ice and stored them at −80°C. We collected coronal brain sections (40 μm) of vSub in PBS containing 0.1% sodium azide and stored them at 4°C until further processing.

We washed free-floating sections three times for 10 min each in PBS and incubated them in reaction buffer (2.4 mM X-gal, 100 mM sodium phosphate, 100 mM sodium chloride, 5 mM EGTA, 2 mM MgCl_2_, 0.2% Triton X-100, 5 mM K_3_FeCN_6_, and 5 mM K_4_FeCN_6_) for 6.5 hours at 37°C with gentle shaking. We washed sections three times for 10 min each in PBS, mounted them onto chrome alum/gelatin-coated slides, and air-dried them. We dehydrated the slides through a graded series of alcohol (30, 60, 90, 95, and 2 × 100% ethanol), cleared them with Citrasolv, and coverslipped the slides with Permount. We captured bright-field images of vSub with a Retiga 2000R CCD camera (QImaging) attached to a Zeiss microscope Axio Scope A1 using a 10× objective. We counted β-gal–expressing nuclei, characterized by blue nuclear staining, using iVision (4.5.0, Biovision Technologies) in sampling areas around vSub injection site (left and right hemispheres) in four coronal sections per rat. We performed image-based quantification of the β-gal–labeled neurons using two independent blind observers (interrater reliability between IF and AS, *r* = 0.99, *P* < 0.01).

### Immunofluorescence double-labeling histochemistry for Fos and β-gal

We double-labeled β-gal with Fos using fluorescent immunohistochemistry as previously described in Caprioli *et al.* (*33*) in two Fos-lacZ transgenic rats exposed to a novel context session for 90 min. We rinsed the sections three times with PBS and incubated them for 2 hours in a blocking buffer containing 4% BSA in PBS with 0.3% PBS-TX. We then incubated all sections for at least 24 hours at 4°C in anti-Fos primary antibody (1:1000; Phospho-Fos, catalog no. 5348S, Cell Signaling Technology, RRID: AB_10557109) and mouse anti–β-gal antibody (1:1000; catalog no. sc65670, lot #A2611, Santa Cruz Biotechnology, RRID: AB_831022) in 4% BSA in 0.3% PBS-TX. Next, we rinsed the sections three times with PBS and incubated them with donkey anti-rabbit Alexa Fluor 488 (1:500; catalog no. A21206, lot #1480470, Invitrogen. RRID: AB_141708) and goat anti-mouse Alexa Fluor 568 (1:500; catalog no. A11004; lot #1419715, Invitrogen. RRID: AB_141371) for 2 hours, followed by three rinses in PBS. We then mounted the sections on chrome alum/gelatin–coated slides and coverslipped them with VECTASHIELD HardSet Antifade Mounting Medium (catalog no. H-1400, Vector Laboratories). We captured fluorescent images of labeled cells using an ORCA Flash 4.0LT (Hamamatsu Photonics) attached to a Zeiss Axio Scope Imager M2 using Micro-Manager (v1.4). We quantified Fos-IR cells or β-gal–IR cells and calculated the percentage of colabeled cells from four sections per rat using the ImageJ.

### Fluorescence-activated cell sorting

We euthanized the rats with isoflurane immediately after the 90-min relapse test. We dissected fresh vSub tissue from 1-mm-thick coronal sections approximately between 5.5 to 6.5 mm posterior to Bregma. We froze the tissue for 30 s in cold isopentane (approximately −40°C) and then stored the tissues in microcentrifuge tubes at −80°C until FACS processing.

We processed the tissue for FACS as described previously for fresh frozen tissue (*45*) with the following modifications: (i) We finely minced the tissue with razor blades on ice and transferred the tissue into 1.5 ml of ice-cold hibernate A (catalog no. HA-if, BrainBits) containing ribonuclease (RNase) inhibitor (1:200; catalog no. 30281-2, Lucigen); (ii) we pooled two-three samples, matched to have similar active lever press responding during the relapse test, which allowed us to increase RNA yield from Fos-positive cells; (iii) we fixed and permeabilized cells by adding the same volume of 100% of cold methanol (−20°C) for 15 min on ice, inverting the tubes every 5 min; and (iv) after collecting the cells by centrifugation (1700*g*, 4 min, 4°C), we resuspended the cells in 0.6 ml of cold PBS + RNase inhibitor and then filtered the cells with 100-μm cell strainers (Falcon brand, BD Biosciences).

We incubated cells with phycoertyhrin (PE)–labeled anti-NeuN antibody (1:500; catalog no. FCMAB317PE, Millipore. RRID: AB_10807694) and Alexa 647–labeled anti–phospho-Fos antibody (1:100; catalog no. 8677, Cell Signaling Technology. RRID: AB_11178518) for 30 min at 4°C and then washed the cells with 0.8 ml of cold PBS. After collecting the cells by centrifugation (1300*g*, 3 min, 4°C), we washed the cells again with 1 ml of cold PBS, followed by centrifugation (1300*g*, 3 min, 4°C), filtered with a 100-μm cell strainer, and resuspended the cells in 0.3 ml of cold PBS + RNase inhibitor for sorting in a FACS Melody cell sorter (BD Biosciences).

As we reported previously (*38*–*40*, *45*), cells can be identified based on the distinct forward and side scatter properties. We excluded duplets based on the forward scatter-H (height) and forward scatter-W (width) scatter signal properties of the cell gate population. From the singlets’ gate, we sorted neurons according to PE (NeuN-immunopositive) and Alexa Fluor 647 (Fos-immunopositive) fluorescence signal. We set the threshold of Alexa Fluor 647 fluorescence signal based on background fluorescence signals of a naïve homecage control group. On the basis of NeuN and Fos immunoreactivity, we sorted Fos-negative/NeuN-positive and Fos-positive/NeuN-positive events (fig. S4D).

### Quantitative PCR

We collected 5000 Fos-negative neurons (Fos-negative-/NeuN-positive) and all Fos-positive neurons (Fos-positive/NeuN-positive) directly into 100 μl of the extraction buffer from a PicoPure RNA isolation kit (catalog no. KIT0204, Applied Biosystems) and lysed the cells by pipetting up and down 10 times followed by incubation for 30 min at 42°C. We extracted RNA according to PicoPure RNA isolation protocol and synthesized single-strand complementary DNA (cDNA) using a SuperScript III first-strand cDNA synthesis kit (catalog no. 18080-051, Invitrogen) according to the manufacturer’s protocol.

We used gene-targeted preamplification of cDNA as described previously (*38*–*40*). Briefly, we used a pooled primer solution of 0.2× concentration of TaqMan ABI primer/probes (20x TaqMan gene expression assay as the stock solution) and 80 nM customized primer sets (table S3). Each cDNA sample (7.5 μl) was mixed with the pooled primer solution (7.5 μl) and 15 μl of 2x TaqMan PreAmp Master Mix (catalog no. 4391128, Applied Biosystems). We preamplified cDNA in an ABI 9700 Thermal Cycler using the following program: 95°C hold for 10 min, denaturation at 90°C for 15 s, and annealing and extension at 60°C for 4 min (14 cycles).

We diluted the preamplified cDNA product seven times before proceeding to qPCR. We performed qPCR in duplicates with a Fam-labeled probe for each target gene and a Vic-labeled probe for the endogenous control gene (NeuN) (*38*–*40*, *45*). We used TaqMan Advanced Fast PCR Master Mix (catalog no. 4444557, Thermo Fisher Scientific) in 7500 Fast TaqMan instrument using the following program: 95°C hold for 20 s, then 40 cycles with denaturation at 95°C for 3 s, and annealing and extension at 60°C for 30 s. We analyzed reactions using the ΔΔ*C*t method with NeuN as the housekeeping gene (*38*–*40*, *45*).

### Self-administration apparatus

We used Med Associates chambers containing two levers located 7.5 to 8 cm above the grid floor on opposing walls. Responding on the active retractable lever activated the infusion pump, while lever presses on the inactive, nonretractable lever had no consequences. We equipped each chamber with a stainless-steel grid floor connected to a shocker (Med Associates ENV-410B).

### General behavior procedure

The experiments included some or all of the following phases: oxycodone self-administration training (14 days), early tests for oxycodone seeking (abstinence day 1), electric barrier–induced abstinence (13 or 16 days), and late tests for oxycodone seeking (abstinence day 15 or 18). We provide details of the different phases for each experiment below.

### Oxycodone self-administration training

We trained the rats to self-administer oxycodone-HCl for 6 hours/day (six 1-hour sessions separated by 10 min) for 14 days. Oxycodone was infused at a volume of 100 μl over 3.5 s at a unit dose of 0.1 mg/kg per infusion. Each session began with illumination of a red houselight that remained on for the entire session, followed 10 s later by the insertion of the active lever. Active lever presses led to oxycodone infusions that were paired with a 20-s tone light cue under a fixed-ratio 1 reinforcement schedule [fixed interval 20-s schedule]. At the end of each 1-hour session, the houselight turned off, and the active lever retracted. We limited oxycodone intake to 15 infusions per hour. We show the training data of experiments 1 to 4 in Fig. 1.

### Electric barrier–induced abstinence

During this phase, oxycodone was available for 2 hours per day for 13 or 16 days. We used the same parameters (oxycodone dose, reinforcement schedule, tone-light cues, etc.) that we used during the training phase. To achieve abstinence, we introduced an electric barrier near the active lever (“shock zone”) (*7*, *76*). We separated the shock zone (two-thirds of the chamber) from a “safe zone” (remaining one-third of the chamber) with a plastic demarcation (Mcmaster-Carr, catalog no. 9852K61). If the rats approached the active lever, then they received a continuous mild footshock (0.1 to 0.4 mA). On the first day, we set the current at 0.0 mA and then gradually increased the intensity to 0.3 mA (0.1-mA increments per day). If a rat did not suppress oxycodone self-administration (<3 infusions per day), then we increased the intensity to 0.4 mA the next day. Before the electric barrier phase, we tested the rats’ sensitivity to footshock (operationally defined as the minimal shock level that causes the withdrawal of the front paw). There were no group or sex differences in shock sensitivity in any of the experiments, assessed with 0.05-mA increments, starting at 0.05 mA, and the values for individual rats ranged from 0.15 to 0.2 mA.

### Forced abstinence

During the forced abstinence phase, we placed the rats in the operant self-administration chamber and left them in the chamber for 2 hours per day without turning on the electric barrier program to account for comparable time spent in the chamber during the electric barrier phase.

### Relapse (incubation) tests

We tested the rats for oxycodone seeking for 30 or 90 min under extinction conditions during early (day 1), late (day 15 or 18) abstinence, or both. During testing, we turned off the electric barrier and removed the plastic demarcation. We gave all rats a 30-min habituation period in the self-administration chamber before the start of the test session to allow them to realize that the barrier is not electrified. Lever presses resulted in the delivery of the oxycodone-paired tone-light cue and activation of the infusion pump but no drug infusions.

#### Experiment 1: vSub Fos expression after a test for incubated oxycodone seeking on day 15

The goal of experiment 1 was to determine whether incubation of oxycodone seeking after electric barrier–induced abstinence is associated with increased Fos expression in vSub after the 90-min day 15 relapse test. The experiment consisted of four phases (Fig. 2A): oxycodone self-administration training (14 days), early tests for oxycodone seeking (abstinence day 1), electric barrier–induced abstinence (13 days), and late tests for oxycodone seeking (abstinence day 15). We used two groups of male and female rats (*n* = 6 to 7 per group) in an experimental design that included the between-subjects factor of test condition (no-test and test). We matched the groups for oxycodone intake during the training and electric barrier phases. On abstinence day 15, all rats experienced a 90-min relapse test, and rats from the test group were perfused immediately after the relapse test. We perfused rats from the no-test group the following day. To verify that incubation had occurred, we compared the number of lever presses during the 30-min day 1 test to the number of lever presses during the first 30 min of the day 15 test.

#### Experiment 2: Effect of muscimol-baclofen vSub inactivation on incubation after electric barrier–induced abstinence

In experiment 1, we found that incubation of oxycodone seeking after electric barrier–induced abstinence was associated with increased Fos expression in vSub. Therefore, the goal of experiment 2 was to determine whether vSub plays a causal role in incubation of oxycodone seeking after electric barrier–induced abstinence. For this purpose, we used the classical muscimol-baclofen inactivation procedure (*25*). The experiment consisted of four phases (Fig. 3A): oxycodone self-administration training (14 days), early tests for oxycodone seeking (abstinence day 1), electric barrier–induced abstinence (13 days), and late tests for oxycodone seeking (abstinence day 15). We used four groups of male and female rats (*n* = 10 to 14 per group) in a mixed experimental design that included the between-subjects factors of abstinence day (1 or 15) and muscimol-baclofen dose (0 and 50 + 50 ng per side) and the within-subjects factor of session time (30, 60, or 90 min). We matched the different groups for total oxycodone infusions during the training phase. We compared number of lever presses on days 1 and 15 tests in rats injected with either saline or muscimol-baclofen into vSub 30 min before the relapse tests on day 1 or 15. We extracted brains after the tests to verify cannula placements.

#### Experiment 3: Effect of muscimol-baclofen vSub inactivation on incubation after forced abstinence

The goal of experiment 3 was to test the specificity of the effect of muscimol-baclofen vSub inactivation on incubated oxycodone seeking after electric barrier–induced abstinence. For this purpose, we determined the effect of vSub vehicle (saline) or muscimol-baclofen (50 + 50 ng per side) injections on incubated (day 15) oxycodone craving after forced abstinence. The experiment consisted of three phases (Fig. 4A): oxycodone self-administration training (14 days), forced abstinence (14 days), and late tests for oxycodone seeking (abstinence day 15). During the forced abstinence phase, we moved the rats from the animal facility to their operant self-administration chamber and left them in the chamber for 2 hours per day without turning on the electric barrier program. We used two groups of male and female rats (*n* = 16 to 18 per group) in a mixed experimental design that included the between-subject factor of muscimol-baclofen dose (0 and 50 + 50 ng per side) and the within-subjects factor of session time (30, 60, or 90 min). We matched the different groups for total oxycodone infusions during the training phase. We compared the number of lever presses on the day 15 relapse test in rats injected with either saline or muscimol-baclofen into the vSub 15 min before testing. We extracted the brains after the test to verify cannula placements.

#### Experiment 4: Effect of Daun02 vSub inactivation on incubation after electric barrier–induced abstinence

In experiment 4, we used the Daun02 inactivation procedure (*26*) to determine whether activated neuronal ensembles in vSub play a causal role in incubated oxycodone seeking after electric barrier–induced abstinence. The experiment consisted of five phases (Fig. 5A): oxycodone self-administration training (14 days), electric barrier–induced abstinence (14 days), induction session (abstinence day 15), electric barrier induced abstinence (2 days), and late tests for oxycodone seeking (abstinence day 18). On the induction session (abstinence day 15), we briefly exposed groups of rats for 15 min to either the oxycodone self-administration context and cues associated with oxycodone injections (lever presses under extinction conditions) to induce relapse-dependent Fos in vSub or a novel context (a plastic bowl with toys) to induce relapse-independent Fos in vSub. Next, 90 min after the induction sessions, when Fos and β-gal expression (*26*) are at their peak, we injected the rats with Daun02 (to inactivate the Fos-positive–activated neurons) or vehicle. Between the induction day and the relapse test on abstinence 18, we conducted two additional electric barrier sessions. We used four groups of rats (*n* = 12 to 17 per group) and analyzed the data of the rats in each induction context (novel context and self-administration context) separately in a mixed experimental design with the between-subjects factor of Daun02 dose (0 and 4 μg per side) and the within-subjects factor of Session time (30, 60, or 90 min). We matched the different groups for total oxycodone intake during the training and electric barrier phases. We compared number of active lever presses on the day 18 relapse test in rats injected with either saline or Daun02 dose (0 and 4 μg per side) into the vSub on abstinence day 15. We perfused the rats after the test, verified cannula placements, and performed Fos and β-gal immunohistochemistry.

#### Experiment 5: Neuronal isolation and molecular phenotyping of vSub Fos-expressing neurons

In experiment 5, we used FACS and qPCR to identify the molecular phenotype of vSub Fos-expressing neuronal ensembles associated with incubated oxycodone seeking after electric barrier–induced abstinence. The experiment consisted of three phases (fig. S4A): oxycodone self-administration training (14 days), electric barrier–induced abstinence (11 days), and late test for oxycodone seeking (abstinence day 15). We used one group of male and female rats (*n* = 13) in an experimental design that included the within-subjects factors of lever (active and inactive) and session time (30, 60, and 90 min) for the relapse test. On abstinence day 15, all rats were exposed to the 90-min relapse test, and we extracted their brains immediately after the relapse test. We then performed FACS and qPCR.

#### Experiment 6: Longitudinal functional connectivity changes in vSub-related circuits

We analyzed the brain images from our recently published study (*18*). These analyses only included male data because we did not test female rats in this study. The experiment consisted of the following general events (Fig. 6A): pretraining fMRI scan, 14 days of oxycodone or food self-administration training, early abstinence relapse test and fMRI scan, 12 days of electric barrier–induced abstinence, and late abstinence relapse test and fMRI scan. The experimental procedures for oxycodone self-administration and electric barrier–induced abstinence (*n* = 22 Sprague-Dawley males) were identical to those described above, except that the voluntary abstinence only lasted 12 days. The experimental procedure for food self-administration and electric barrier–induced abstinence (*n* = 22 males) was the same as the oxycodone self-administration, except that lever presses resulted in the delivery of five pellets 1 s apart (TestDiet, catalog no. 1811155; 12.7% fat, 66.7% carbohydrate, and 20.6% protein). Additional details about the fMRI experiment can be found in (*18*). The goal of our fMRI analyses was to determine whether longitudinal vSub-related functional connectivity changes induced by oxycodone (or palatable food) self-administration and subsequently electric barrier–induced abstinence would predict incubation of oxycodone (or food) seeking, operationally defined as the “incubation score”: active lever presses during the relapse tests during late abstinence (day 15) minus lever presses during the early abstinence (day 1) (Fig. 6A).

As discussed by Fredriksson *et al.* (*18*), because of potential carry-over performance-disrupting effects of the imaging procedure (which requires an extensive duration of anesthesia) on operant responding 1 day after the imaging session, we performed the early and late abstinence scans 1 day after the relapse tests (days 2 and 15). It is unlikely that the experience of acute relapse tests, rather than self-administration or voluntary abstinence experience, accounts for the functional connectivity changes reported in that study and the current analyses of the images from that study with an initial seed in vSub [for more details, see Discussion section of Fredriksson *et al.* (*18*)].

### Statistical analyses

#### Behavioral and immunohistochemistry data analyses

We analyzed the behavioral and immunohistochemistry data with RM-ANOVAs, RM-ANCOVAs, ANOVAs, and ANCOVAs (inactive lever as a covariate) using Statistical Package for the Social Sciences (SPSS) (version 24, GLM procedure). We followed significant main effects and interactions (*P* < 0.05) with post hoc tests [univariate ANOVAs or Fisher's Protected Least Significant Differences (PLSD)]. We describe the different between- and within-subjects factors for the different analyses in Results. Because the multifactorial ANCOVAs yielded multiple main and interaction effects, we only report significant effects that are critical for data interpretation. For clarity, we indicate the results of post hoc analyses with asterisks in the figures but do not describe them in Results. For a complete reporting of the statistical analyses, see table S1, and for individual data of the bar graphs described in Figs. 2 to 5, see fig. S1.

#### qPCR data analyses

We analyzed the qPCR data with linear mixed effects modeling (*77*) using JMP 16. Specifically, we used cell type (nominal, Fos negative versus Fos positive) as a fixed within-subjects factor and sample number as a random factor. For a complete reporting of the statistical analyses, see table S2.

#### fMRI data analyses

The flowchart of the fMRI analyses is shown in fig. S3. We preprocessed fMRI data with a standard pipeline, including skull stripping, motion correction, coregistration to a template, noise component removal, temporal filtering, spatial smoothing, nuisance covariance regression for respiration, head motion, and body temperature. We excluded 7 of the 44 rats from the analyses because their head motion exceeded a standard threshold (*78*). Next, we delineated separate seed regions from the right and left vSub (Fig. 6B) based on a rat atlas (*79*). The fMRI data analyses were done as previously described by Fredriksson *et al.* (*18*). Specifically, we computed voxel-wise Pearson’s correlation coefficients between the time course of the seeds and all brain voxels and converted them to *z* scores (achieving an approximate normal distribution) to evaluate functional connectivity between brain regions.

We conducted a voxel-wise reward [food (*n* = 19), oxycodone (*n* = 18)] × time (pretraining, early abstinence) ANOVAs to assess differential changes of functional connectivity associated with oxycodone or food self-administration (self-administration–related connectivity changes). We then conducted a voxel-wise reward [food (*n* = 19), oxycodone (*n* = 18)] × time (early abstinence, late abstinence) ANOVA to examine the effect of electric barrier–induced abstinence on functional connectivity in the two groups (voluntary abstinence-related connectivity changes). In the voxel-wise ANOVA, we corrected for multiple comparisons and used corrected *P*_corr_ < 0.025 [with uncorrected *P* < 0.005 and cluster size of >10 based on Monte Carlo simulation in Analysis of Functional NeuroImages (AFNI)] as a threshold to determine voxels that are significant in the analyses.

We extracted functional connectivity signals from brain regions showing significant effects of interaction in ANOVA results. We then correlated functional connectivity changes from pretraining to early abstinence, functional connectivity changes from early abstinence to late abstinence, and functional connectivity at early abstinence with the incubation score (active lever presses during the relapse tests on abstinence day 15 minus lever presses on day 1; Fig. 6A). For a complete reporting of the correlation analyses, see tables S4 and S5.

## Notes

View/request a protocol for this paper from *Bio-protocol*.
