# Supplementary material for: Role of ventral subiculum neuronal ensembles in incubation of oxycodone craving after electric barrier–induced voluntary abstinence
Source: Sci Adv. 2023 Jan 11;9(2):eadd8687. doi: 10.1126/sciadv.add8687 (PMC9833671; doi:10.1126/sciadv.add8687)
Supplement: Supplementary file 1 — Tables S1 to S5 Figs. S1 to S4 [file sciadv.add8687_sm.pdf]

Science Advances
AAAS

Supplementary Materials for

Role of ventral subiculum neuronal ensembles in incubation of oxycodone craving after electric barrier–induced voluntary abstinence

Ida Fredriksson *et al.*

Corresponding author: Ida Fredriksson, ida.fredriksson@nih.gov; Yavin Shaham, yavin.shaham@nih.gov

Sci. Adv. **9**, eadd8687 (2023)
DOI: 10.1126/sciadv.add8687

The PDF file includes:

Tables S1 to S5
Figs. S1 to S4
Legend for data S1

Other Supplementary Material for this manuscript includes the following:

Data S1

Table S1. Statistical analysis for the behavioral results (SPSS GLM repeated-measures module). Partial Eta^2 = proportion of explained variance. For ANCOVAs, the covariate is inactive lever presses. RM, repeated measures; SA, self-administration; M+B, muscimol-baclofen; vSub, ventral subiculum

Exp. 1. vSub Fos expression after a test for incubated oxycodone craving on day 15

Figure number	Factor name	F-value	*p*-value	Partial Eta^2
Figure 1A. Self-administration training<br>Infusions<br>RM-ANOVA<br>Within-subjects factor: Session	Session (1-14) within-subjects	$F_{13,156}$ = 10.5	<0.001*	0.466
Figure 1A. Self-administration training<br>Active lever presses<br>RM-ANOVA<br>Within-subjects factors: Session, Lever	Session (1-14) within-subjects<br>Lever (Active, Inactive) within subjects<br>Lever X Session interaction	$F_{13,156}$ = 1.8<br>$F_{1,12}$ = 7.7<br>$F_{13,156}$ = 1.9	0.053<br>0.017*<br>0.030*	0.128<br>0.390<br>0.139
Figure 1A. Electric barrier<br>Infusions<br>RM-ANOVA<br>Within-subjects factor: Session	Session (1-13) within-subjects	$F_{12,144}$ = 49.3	<0.001*	0.804
Figure 1A. Electric barrier<br>Active lever presses<br>RM-ANOVA<br>Within-subjects factors: Session, Lever	Session (1-13) within-subjects<br>Lever (Active, Inactive) within subjects<br>Lever X Session interaction	$F_{12,144}$ = 5.4<br>$F_{1,12}$ = 0.02<br>$F_{12,144}$ = 8.0	<0.001*<br>0.898<br><0.001*	0.309<br>0.001<br>0.401
Figure 2B. Relapse tests days 1 and 15 (Total 30 min)<br>Active lever presses<br>RM-ANCOVA<br>Within-subjects factor: Abstinence day<br>Covariates: Inactive day 1, Inactive day 15 (30 min)	Abstinence day (1,15) within-subjects	$F_{1,10}$ = 6.2	0.032*	0.384
Figure 2B. Relapse tests days 1 and 15 (30 min time course)<br>Active lever presses<br>RM-ANCOVA<br>Within-subjects factors: Abstinence day, Session time<br>Covariates: Inactive day 1, Inactive day 15 (30 min)	Abstinence day (1,15) within-subjects<br>Session time (10, 20, 30) within-subjects<br>Abstinence day X Session time interaction	$F_{1,10}$ = 6.2<br>$F_{2,20}$ = 17.1<br>$F_{2,20}$ = 7.0	0.032*<br><0.001*<br>0.005*	0.384<br>0.631<br>0.412
Figure 2C. Fos immunohistochemistry<br>vSub Fos Counts<br>One-way ANOVA<br>Between-subjects factor: Test condition	Test condition (No Test, Test) between-subjects	$F_{1,11}$ = 36.3	<0.001*	0.768
Figure 2D. Fos immunohistochemistry of vSub subregions<br>vSub Fos Counts<br>Mixed ANOVA<br>Between-subjects factor: Test condition<br>Within-subjects factor: Subdivision<br>Within-subjects factor: subregion	Test condition (No Test, Test) between-subjects<br>Subregion (Anterior, Posterior) within-subjects<br>Location (Distal, Proximal) within-subjects<br>Location x Subregion<br>Location x Test condition<br>Test condition x Subregion<br>Location x Subregion x Test condition	$F_{1,11}$ = 318.4<br>$F_{1,11}$ = 7.5<br>$F_{1,11}$ = 64.8<br>$F_{1,11}$ = 9.6<br>$F_{1,11}$ = 7.0<br>$F_{1,11}$ = 6.2<br>$F_{1,11}$ = 0.3	<0.001*<br>0.019*<br><0.001*<br>0.010*<br>0.023*<br>0.030*<br>0.594	0.967<br>0.407<br>0.855<br>0.465<br>0.389<br>0.360<br>0.027

Exp. 2. Effect of muscimol-baclofen vSub inactivation on incubation after electric barrier-induced abstinence

Figure number	Factor name	F-value	*p*-value	Partial Eta^2
Figure 1B. Self-administration training<br>Infusions<br>RM-ANOVA<br>Within-subjects factor: Session	Session (1-14) within-subjects	$F_{13,611}$ = 41.2	<0.001*	0.467
Figure 1B. Self-administration training<br>Active lever presses<br>RM-ANOVA<br>Within-subjects factors: Session, Lever	Session (1-14) within-subjects<br>Lever (Active, Inactive) within subjects<br>Lever X Session interaction	$F_{13,611}$ = 16.3<br>$F_{1,47}$ = 134.6<br>$F_{13,611}$ = 17.9	<0.001*<br><0.001*<br><0.001*	0.257<br>0.741<br>0.276
Figure 1B. Electric barrier<br>Infusions<br>RM-ANOVA<br>Within-subjects factor: Session	Session (1-12) within-subjects	$F_{11,220}$= 30.5	<0.001*	0.604
Figure 1B. Electric barrier<br>Active lever presses<br>RM-ANOVA<br>Within-subjects factors: Session, Lever	Session (1-12) within-subjects<br>Lever (Active, Inactive) within subjects<br>Lever X Session interaction	$F_{11,220}$ = 15.9<br>$F_{1,20}$ = 0.1<br>$F_{11,220}$ = 26.5	<0.001*<br>0.73<br><0.001*	0.442<br>0.006<br>0.57
Figure 3B. Relapse tests days 1 and 15 (Total 90 min)<br>Active lever presses<br>Two-way ANCOVA<br>Between-subjects factors: Abstinence day, M+B dose<br>Covariate: Inactive (90 min)	Abstinence day (1,15) between-subjects<br>M+B dose (0, 50+50ng/side) between-subjects<br>Abstinence day X M+B dose interaction	$F_{1,43}$ = 43.4<br>$F_{1,43}$ = 10.5<br>$F_{1,43}$ = 7.3	<0.001*<br>0.002*<br>0.01*	0.502<br>0.197<br>0.144
Figure 3B. Relapse tests days 1 and 15 (90 min time course)<br>Active lever presses<br>Mixed ANCOVA<br>Between-subjects factors: Abstinence day, M+B dose<br>Within-subjects factor: Session time<br>Covariate: Inactive (90 min)	Abstinence day (1,15) between-subjects<br>M+B dose (0, 50+50ng/side) between-subjects<br>Abstinence day X M+B dose interaction<br><br>Session time (30, 60, 90) within-subjects<br>Session time X Abstinence day interaction<br>Session time X M+B dose interaction<br>Session time X Abstinence day X M+B dose interaction	$F_{1,43}$ = 43.3<br>$F_{1,43}$ = 10.5<br>$F_{1,43}$ = 7.3<br><br>$F_{2,86}$ = 62.7<br>$F_{2,86}$ = 14.4<br>$F_{2,86}$ = 4.4<br>$F_{2,86}$ = 2.7	<0.001*<br>0.002*<br>0.01*<br><br><0.001*<br><0.001*<br>0.015*<br>0.073	0.502<br>0.197<br>0.145<br><br>0.593<br>0.251<br>0.093<br>0.059

Exp. 3. Effect of muscimol-baclofen vSub inactivation on incubation after forced abstinence

Figure number	Factor name	F-value	*p*-value	Partial Eta^2
Figure 1C. Self-administration training<br>Infusions<br>RM-ANOVA<br>Within-subjects factor: Session	Session (1-14) within-subjects	$F_{13,429}$ = 20.0	<0.001*	0.377
Figure 1C. Self-administration training<br>Active Lever presses<br>RM-ANOVA<br>Within-subjects factors: Session, Lever	Session (1-14) within-subjects<br>Lever (Active, Inactive) within subjects<br>Lever X Session interaction	$F_{13,429}$ = 4.8<br>$F_{1,33}$ = 48.6<br>$F_{13,429}$ = 5.4	<0.001*<br><0.001*<br><0.001*	0.128<br>0.596<br>0.140
Figure 4B. Relapse test day 15 (Total 90 min)<br>Active lever presses<br>One-Way ANCOVA<br>Between-subjects factor: M+B dose<br>Covariate: Inactive (90 min)	M+B dose (0, 50+50ng/side) between-subjects	$F_{1,31}$ = 0.2	0.644	0.007

Figure 4B. Relapse test day 15 (90 min time course)<br>Active lever presses<br>Mixed ANCOVA<br>Between-subjects factor: M+B dose<br>Within-subjects factor: Session time<br>Covariate: Inactive (90 min)	M+B dose (0, 50+50ng/side) between-subjects<br>Session time (30, 60, 90) within-subjects<br>Session time X M+B dose interaction	$F_{1,31} = 0.2$<br>$F_{2,62} = 30.2$<br>$F_{2,62} = 0.4$	0.644<br><0.001*<br>0.671	0.007<br>0.494<br>0.013

Exp. 4. Effect of Daun02 vSub inactivation on incubation after electric barrier-induced abstinence

Figure number	Factor name	F-value	*p*-value	Partial Eta^2
Figure 1D. Self-administration training<br>Infusions<br>RM-ANOVA<br>Within-subjects factor: Session	Session (1-14) within-subjects	$F_{13,754} = 85.2$	<0.001*	0.595
Figure 1D. Self-administration training<br>Active lever presses<br>RM-ANOVA<br>Within-subjects factors: Session, Lever	Session (1-14) within-subjects<br>Lever (Active, Inactive) within subjects<br>Lever X Session interaction	$F_{13,754} = 14.8$<br>$F_{1,58} = 55.8$<br>$F_{13,754} = 14.6$	<0.001*<br><0.001*<br><0.001*	0.203<br>0.490<br>0.201
Figure 1D. Electric barrier<br>Infusions<br>RM-ANOVA<br>Within-subjects factor: Session	Session (1-13) within-subjects	$F_{12,696} = 139.1$	<0.001*	0.706
Figure 1D. Electric barrier<br>Active lever presses<br>RM-ANOVA<br>Within-subjects factors: Session, Lever	Session (1-13) within-subjects<br>Lever (Active, Inactive) within subjects<br>Lever X Session interaction	$F_{12,696} = 27.6$<br>$F_{1,58} = 11.4$<br>$F_{12,696} = 33.5$	<0.001*<br>0.001*<br><0.001*	0.323<br>0.164<br>0.366
SA context Induction session day 15 (15 min)	Daun02 dose (0, 4µg/side) between-subjects	$F_{1,23} = 0.02$	0.896	0.001
Figure 5B. SA context Relapse test day 18 (Total 90 min)<br>Active lever presses<br>One-Way ANCOVA<br>Between-subjects factor: Daun02 dose<br>Covariate: Inactive (90 min)	Daun02 dose (0, 4µg/side) between-subjects	$F_{1,23} = 4.5$	0.044*	0.165
Figure 5B. SA context Relapse tests day 18 (90 min time course)<br>Active lever presses<br>Mixed ANCOVA<br>Between-subjects factor: Daun02 dose<br>Within-subjects factor: Session time<br>Covariate: Inactive (90 min)	Daun02 dose (0, 4µg/side) between-subjects<br>Session time within-subjects<br>Session time X Daun02 dose interaction	$F_{1,23} = 4.5$<br>$F_{2,46} = 20.9$<br>$F_{2,46} = 1.1$	0.044*<br><0.001*<br>0.332	0.165<br>0.476<br>0.047
Figure 5C. SA context vSub Fos-Xgal immunohistochemistry<br>Fos and Xgal counts<br>One-way ANOVA<br>Between-subjects factor: Daun02 dose	Fos: Daun02 dose (0, 4µg/side) between-subjects<br>Xgal: Daun02 dose (0, 4µg/side) between-subjects	$F_{1,24} = 50.3$<br>$F_{1,24} = 33.1$	<0.001*<br><0.001*	0.677<br>0.580

Figure 5B. Novel context<br>Relapse test day 18 (Total 90 min)<br><u>Active lever presses</u><br>One-Way ANCOVA<br>Between-subjects factor: Daun02 dose<br>Covariate: Inactive (90 min)	Daun02 dose (0, 4µg/side) between-subjects	$F_{1,30}$ = 0.08	0.779	0.003
Figure 5B. Novel context<br>Relapse tests day 18 (90 min time course)<br><u>Active lever presses</u><br>Mixed ANCOVA<br>Between-subjects factor: Daun02 dose<br>Within-subjects factor: Session time<br>Covariate: Inactive (90 min)	Daun02 dose (0, 4µg/side) between-subjects<br>Session time within-subjects<br>Session time X Daun02 dose interaction	$F_{1,30}$ = 0.08<br>$F_{2,60}$ = 39.1<br>$F_{2,60}$ = 0.8	0.779<br><0.001*<br>0.46	0.003<br>0.566<br>0.026
Figure 5C. Novel context vSub Fos-Xgal immunohistochemistry<br><u>Fos and Xgal counts</u><br>One-way ANOVA<br>Between-subjects factor: Daun02 dose	Fos: Daun02 dose (0, 4µg/side) between-subjects<br>Xgal: Daun02 dose (0, 4µg/side) between-subjects	$F_{1,31}$ = 0.5<br>$F_{1,31}$ = 0.3	0.491<br>0.618	0.015<br>0.008

Exp. 5. Molecular phenotyping of vSub Fos-cells with FACS+qPCR (Behavioral results)

Figure number	Factor name	F-value	*p*-value	Partial Eta^2
Figure S4B. Self-administration training<br><u>Infusions</u><br>RM-ANOVA<br>Within-subjects factor: Session	Session (1-14) within-subjects	$F_{13,156}$ = 5.6	<0.001*	0.316
Figure S4B. Self-administration training<br><u>Active lever presses</u><br>RM-ANOVA<br>Within-subjects factors: Session, Lever	Session (1-14) within-subjects<br>Lever (Active, Inactive) within subjects<br>Lever X Session interaction	$F_{13,156}$ = 2.3<br>$F_{1,12}$ = 10.2<br>$F_{13,156}$ = 2.5	0.010*<br>0.008*<br>0.004*	0.158<br>0.459<br>0.173
Figure S4B. Electric barrier<br><u>Infusions</u><br>RM-ANOVA<br>Within-subjects factor: Session	Session (1-11) within-subjects	$F_{10,120}$ = 36.5	<0.001*	0.752
Figure S4B. Electric barrier<br><u>Active lever presses</u><br>RM-ANOVA<br>Within-subjects factors: Session, Lever	Session (1-11) within-subjects<br>Lever (Active, Inactive) within-subjects<br>Lever X Session interaction	$F_{10,120}$ = 7.1<br>$F_{1,12}$ = 0.11<br>$F_{10,120}$ = 12.0	<0.001*<br>0.741<br><0.001*	0.372<br>0.009<br>0.500
Figure S4C. SA context<br>Relapse tests day 15 (90 min time course)<br><u>Active lever presses</u><br>RM-ANOVA<br>Within-subjects factor: Session time, Lever	Session time within-subjects<br>Lever (Active, Inactive) within-subjects<br>Session time X Leve interaction	$F_{2,24}$ = 36.0<br>$F_{1,12}$ = 64.9<br>$F_{2,24}$ = 36.5	<0.001*<br><0.001*<br><0.001*	0.750<br>0.844<br>0.752

Table S2. Statistical analysis for the qPCR results. Analyses are with linear mixed effects modeling with cell type (nominal) as a fixed within-subjects factor and sample number as a random factor.

Exp. 5. Molecular phenotyping of vSub Fos-positive cells with FACS+qPCR (qPCR results). Fold changes are relative to Fos-negative cells, Mean±SEM)

Gene	Fold change	Linear mixed-effects modeling in JMP
**Acetylcholine-related genes**		
Chrna2	2.4±0.6	$F_{1,1.1024}$=834.4, p=0.02*
Chrna3	0.4±0.2	$F_{1,5.314}$=12.3, p=0.016*
Chrna4	0.9±0.4	$F_{1,4.449}$=0.34, p=0.59
Chrna5	1.7±0.9	$F_{1,4.218}$=1.1, p=0.35
Chrna7	1.4±0.2	$F_{1,4.94}$=3.0, p=0.14
Chrm1	0.9±0.1	$F_{1,4.84}$=0.94, p=0.38
Chrm2	0.7±0.2	$F_{1,3.377}$=0.61, p=0.48
Chrm3	0.9±0.1	$F_{1,5.05}$=0.20, p=0.67
Chrm4	1.2±0.4	$F_{1,5.207}$=0.22, p=0.66
Chrm5	0.9±0.1	$F_{1,4.422}$=1.5, p=0.28
**Dopamine-related genes**		
Drd1	0.8±0.1	$F_{1,3.803}$=1.5, p=0.28
Drd2	3.7±1.2	$F_{1,4.884}$=5.0, p=0.071
**GABA-related genes**		
Gabra1	0.8±0.2	$F_{1,5.015}$=1.4, p=0.29
Gabra3	2.8±0.5	$F_{1,4.857}$=10.5, p=0.024
Gabra5	1.2±0.3	$F_{1,4.861}$=0.64, p=0.46
Gabbr2	0.8±0.2	$F_{1,4.431}$=1.3, p=0.32
Gabrg2	0.9±0.1	$F_{1,5.26}$=0.12, p=0.75
**Glutamate-related genes**		
Gria1	0.9±0.1	$F_{1,4.31}$=7.7, p=0.046
Gria2	1.0±0.1	$F_{1,4.627}$=0.01, p=0.92
Gria3	1.4±0.1	$F_{1,4.853}$=7.1, p=0.046
Gria4	0.8±0.1	$F_{1,4.204}$=12.9, p=0.021
Grin1	1.1±0.1	$F_{1,4.918}$=0.62, p=0.47
Grin2a	0.9±0.1	$F_{1,5.127}$=0.49, p=0.52
Grin2b	0.9±0.1	$F_{1,4.886}$=0.34, p=0.59
Grm1	1.1±0.2	$F_{1,4.813}$=0.44, p=0.54
Grm2	0.7±0.2	$F_{1,5.017}$=6.3, p=0.053
Grm3	1.4±0.4	$F_{1,5.215}$=1.3, p=0.31
Grm4	2.4±0.8	$F_{1,2.333}$=11.8, p=0.06
Grm5	1.0±0.2	$F_{1,4.954}$=0.00, p=1.0
**Immediate early genes**		
Fos	6.0±2.7	F(1,5.025)=5.068, p=0.074
Arc	5.2±1.0	F(1,5.066)=21.500, p=0.006*
**Opioid-related genes**		
Oprm1	2.3±0.1	$F_{1,4.442}$=18.446, p=0.01
Oprd1	0.8±0.1	$F_{1,5.238}$=1.3, p=0.3
Opcml	1.1±0.3	$F_{1, 4.741}$=0.20, p=0.671

Table S3. Primer/probe sequences used for qPCR of FACS-isolated neurons

Gene	TaqMan probe	Forward primer	Reverse primer
NeuN	CACTCCAACAGCGTGAC	GGCCCCTGGCAGAAAGTAG	TTCCCCCTGGTCCTTCTGA
Fos	Rn00487426_g1		
Arc	Rn00571208_g1		
Gria1	Rn00709588_m1		
Gria2	Rn00568514_m1		
Gria3	Rn00583547_m1		
Gria4	Rn00568544_m1		
Grin1	Rn01436038_m1		
Grin2a	Rn00561341_m1		
Grin2b	Rn00680474_m1		
Grm1	Rn01440619_m1		
Grm2	Rn01447672_m1		
Grm3	Rn01755349_m1		
Grm4	Rn01428450_m1		
Grm5	Rn00690337_m1		
Gabra1	Rn00788315_m1		
Gabra3	Rn00567055_m1		
Gabra5	Rn00568803_m1		
Gabbr2	Rn00582550_m1		
Gabrag2	Rn01464079_m1		
Drd1	Rn03062203_s1		
Drd2	Rn00561126_m1		
Oprm1	Rn00565144_m1		
Oprd1	Rn07310941_m1		
Opcml	Rn00587759_m1		
Chrna2	Rn00591542_m1		
Chrna3	Rn00583820_m1		
Chrna4	Rn00577436_m1		
Chrna5	Rn00567155_m1		
Chrna7	Rn00563223_m1		
Chrm1	Rn00589936_m1		
Chrm2	Rn02532311_s1		
Chrm3	Rn00560986_s1		
Chrm4	Rn01512605_s1		
Chrm5	Rn02758749_s1		

Table S4. Summary of Pearson *r* correlations between self-administration- and voluntary abstinence-related functional connectivity changes and the incubation score for the data described in Figures 6-7. * p-value < 0.05; ** p-value < 0.01.

Figure number	Seed area	Target area	Food	Oxycodone
Figure 6B. Self-administration phase	Right vSub	Cingulate cortex	r = -0.16	r = -0.10
		Dorsal hippocampus	r = -0.16	r = -0.31
Figure 7B. Self-administration phase	[Right vSub] Dorsal hippocampus	Medial orbital frontal cortex	r = -0.40	r = 0.27
		Sensory cortex	r = -0.17	r = 0.21
		Dorsal striatum	r = -0.05	r = 0.36
		Thalamus	r = -0.29	r = 0.02
		Dorsal hippocampus	r = -0.05	r = 0.19
		Ventral subiculum	r = -0.27	r = -0.30
Figure 6C. Electric barrier-induced abstinence phase	Right vSub	Retrosplenial cortex	r = 0.31	r = -0.50*

Table S5. Summary of Pearson *r* correlations between the functional connectivity at early abstinence (day 2) and the incubation score for the data described in main text Figures 6-7. * p-value < 0.05; ** p-value < 0.01.

Figure number	Seed area	Target area	Food	Oxycodone
Figure 6D (left). Self-administration phase	Right vSub	Cingulate cortex	r = 0.06	r = -0.27
		Dorsal hippocampus	r = 0.15	r = -0.56*
Figure 7C. Self-administration phase	[Right vSub] Dorsal hippocampus	Medial orbital frontal cortex	r = -0.16	r = 0.47*
		Sensory cortex	r = -0.24	r = 0.54*
		Dorsal striatum	r = -0.008	r = 0.61**
		Thalamus	r = -0.20	r = 0.11
		Dorsal hippocampus	r = -0.12	r = 0.39
		Ventral subiculum	r = 0.08	r = -0.59**
Figure 6D (right). Electric barrier-induced abstinence phase	Right vSub	Retrosplenial cortex	r = -0.04	r = -0.45*

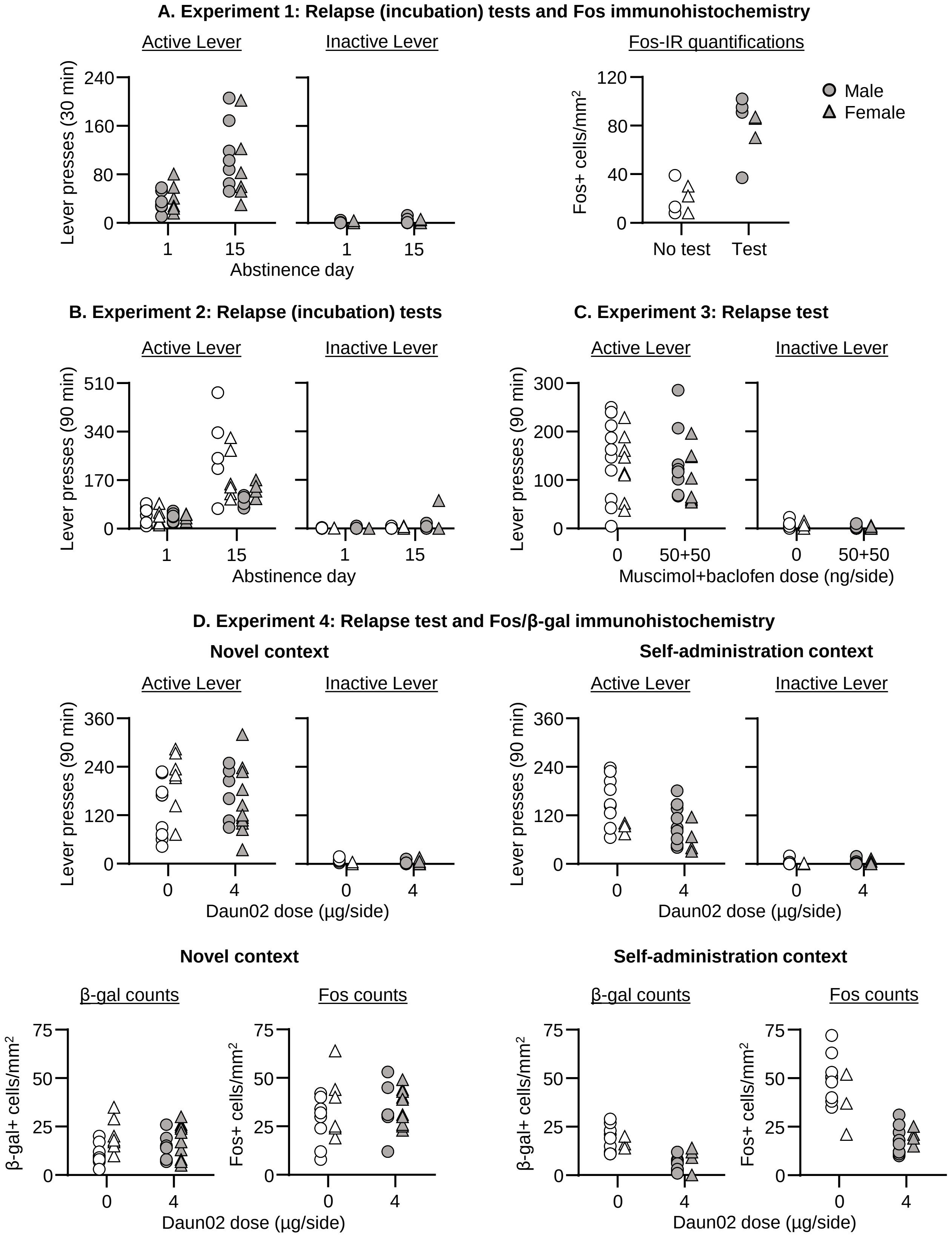

Figure S1. (A-C) Number of active and inactive lever presses during the relapse tests of individual rats in Exp. 1-3, and Fos-positive cells (counts/mm^2) of individual rats in Exp. 1. **(D)** Number of active and inactive lever presses during the relapse tests and number of β-gal-positive or Fos-positive cells (counts/mm^2) of individual rats in Exp. 4. β-gal+, β-gal-positive; Fos+, Fos-positive.

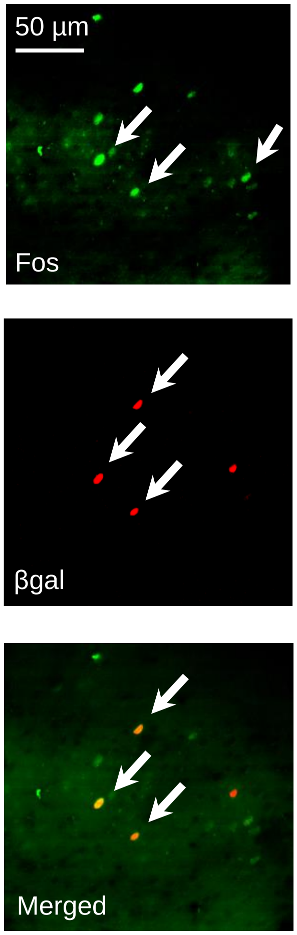

Figure S2. Representative images of Fos-positive, β-gal-positive, and Fos-positive β-gal-positive double labeling cells in vSub at 40X magnification in *c-fos–lacZ* transgenic rats exposed to a novel context (n=2). Fos expression (green-labeled nuclei), β-gal expression (red-labeled nuclei), and nuclei double-labeled for both β-gal and Fos appear yellow to orange in the merged image panel and indicate co-localization of β-gal and Fos proteins.

Figure S3. Flowchart of the image analyses

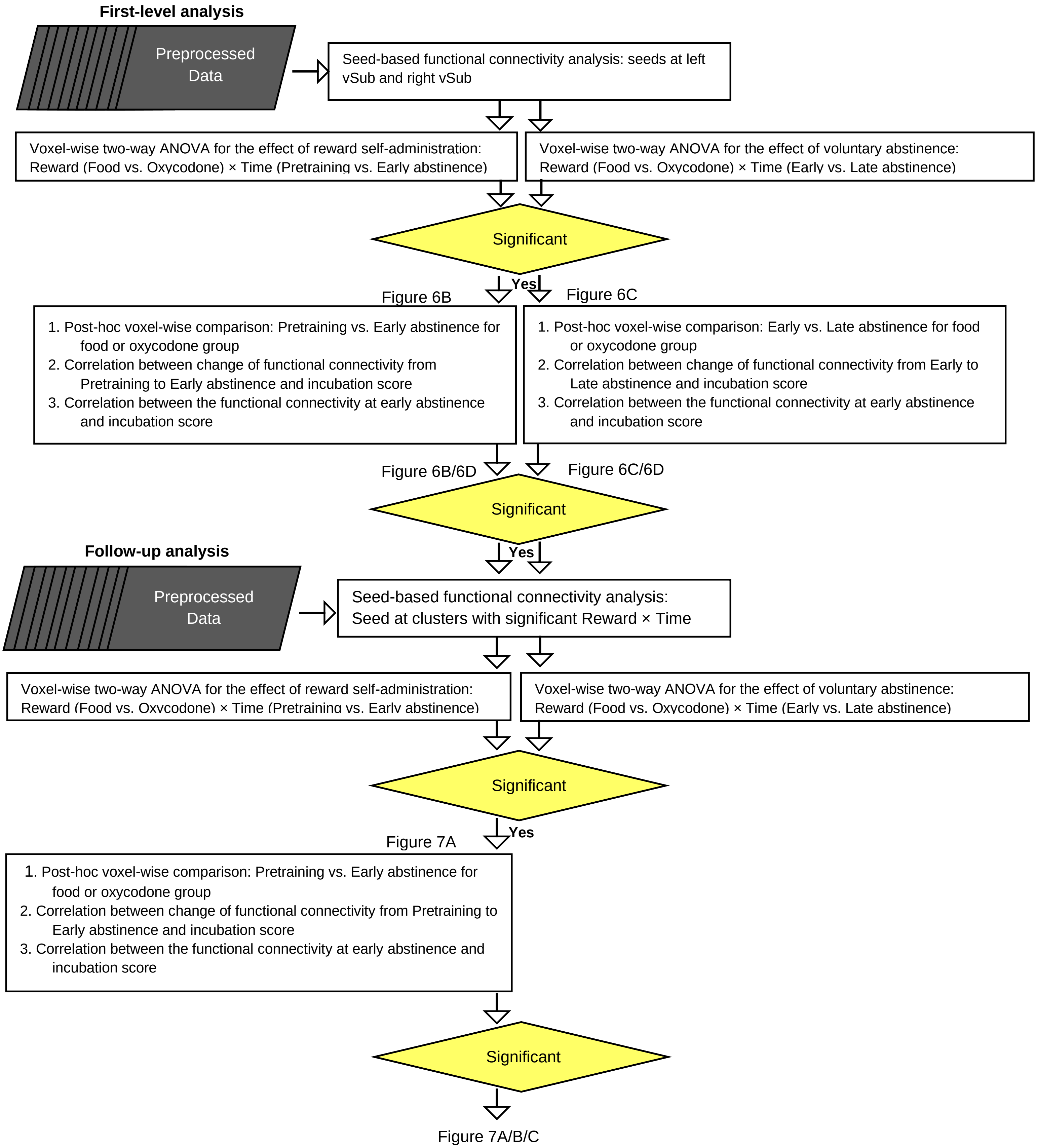

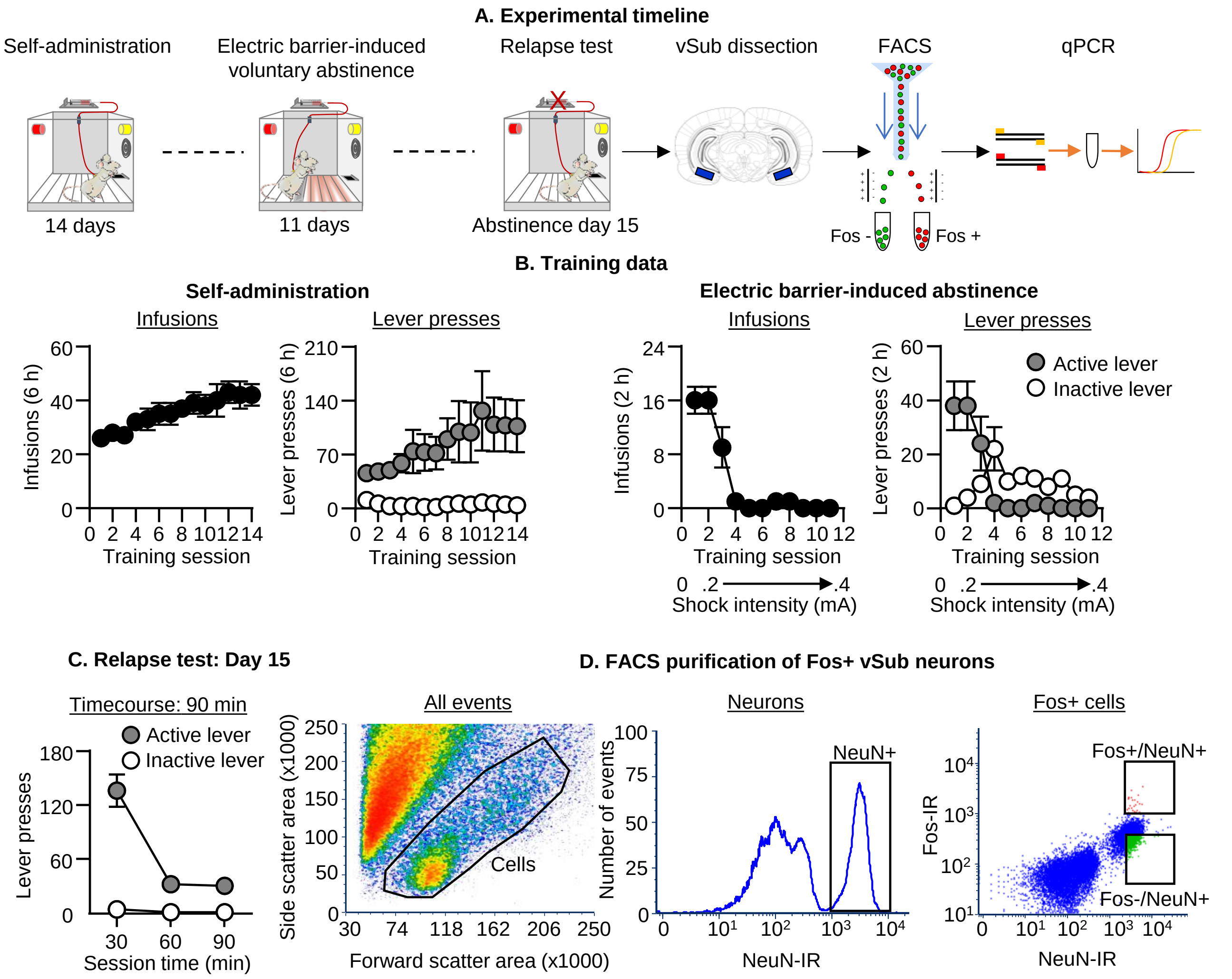

Figure S4. *Behavioral data of Exp. 5 and the FACS procedure.* (**A**) Timeline of Exp. 5. (**B**) Left: Self-administration training. Mean±SEM number of infusions and active and inactive lever presses during the training phase (n=13). Right: Electric barrier-induced abstinence. Mean±SEM number of infusions and active and inactive lever presses during the electric barrier phase. (**C**) Relapse (incubation) test: Mean±SEM number of active and inactive lever presses during the 90-min day 15 test session. (**D**) FACS purification of Fos-positive vSub neurons: *All events (Left):* Representative density plot (red is most dense and blue is least dense) based on their forward scatter (X-axis, cell size) and side scatter (Y-axis, granularity) properties. The “Cells” gate, marked in black, represents ~8% of total events. *Neurons (Middle):* Representative histogram shows 35% Phycoertyhrin (PE)-labeled NeuN-positive events (neurons) within “Cells” gate after duplet exclusion (not shown). *Sorting gates (Right):* Representative dot plot shows PE-(NeuN, x-axis) and Alexa Fluor 647-(Fos, y-axis) fluorescence intensity. Gates show Fos-positive/NeuN-positive events (activated neurons, red dots) and Fos-negative/NeuN-positive events (nonactivated neurons, green dots) collected for qPCR. Fos+, Fos-positive; NeuN+, NeuN-positive.

Data S1. *Individual data of the behavioral part of the study, immunohistochemistry, and the qPCR.* All the raw data for Exp. 1-5 are provided in Data S1.
